# T Cell Response in Ischemic Stroke: From Mechanisms to Translational Insights

**DOI:** 10.3389/fimmu.2021.707972

**Published:** 2021-07-15

**Authors:** Dianhui Zhang, Jiaxin Ren, Yun Luo, Qianyan He, Ruoyu Zhao, Junlei Chang, Yi Yang, Zhen-Ni Guo

**Affiliations:** ^1^ Stroke Center, Department of Neurology, First Affiliated Hospital of Jilin University, Changchun, China; ^2^ Department of Rehabilitation Medicine, First Affiliated Hospital of Gannan Medical University, Ganzhou, China; ^3^ Shenzhen Institute of Advanced Technology, Chinese Academy of Sciences, Shenzhen, China; ^4^ Neuroscience Center, Department of Neurology, First Affiliated Hospital of Jilin University, Changchun, China

**Keywords:** T cells, ischemic stroke, post-stroke inflammation, stroke therapy, immune responses

## Abstract

Ischemic stroke, caused by a sudden disruption of blood flow to the brain, is a leading cause of death and exerts a heavy burden on both patients and public health systems. Currently available treatments for ischemic stroke are very limited and are not feasible in many patients due to strict time windows required for their administration. Thus, novel treatment strategies are keenly required. T cells, which are part of the adaptive immune system, have gained more attention for its effects in ischemic stroke. Both preclinical and clinical studies have revealed the conflicting roles for T cells in post-stroke inflammation and as potential therapeutic targets. This review summarizes the mediators of T cell recruitment, as well as the temporal course of its infiltration through the blood-brain-barrier, choroid plexus, and meningeal pathways. Furthermore, we describe the mechanisms behind the deleterious and beneficial effects of T cells in the brain, in both antigen-dependent and antigen-independent manners, and finally we specifically focus on clinical and preclinical studies that have investigated T cells as potential therapeutic targets for ischemic stroke.

## Introduction

Stroke is the second-leading cause of death and disability worldwide (1–4). Ischemic stroke is the most common stroke type, accounting for 62% of all stroke incidents in 2019 (5, 6). In the acute phase, clinical treatments of ischemic stroke center on recanalization therapies, which restore blood flow to the infarct area and rescue salvageable tissues, and thus promote the recovery of neurological functions (7). FDA-approved recanalization therapies are currently limited to intravenous thrombolysis with recombinant tissue-type plasminogen activator (rtPA) and mechanical thrombectomy (8, 9). However, only a small group of patients can receive these treatments because many patients have missed the strict time window required at the time of admission or diagnosis (10). Therefore, novel treatment strategies for ischemic stroke are urgently needed.

Under homeostatic conditions, immune responses in the brain are rigorously regulated. Immune cells, other than central nervous system (CNS) resident microglia, are largely absent from the parenchyma. Together with the isolation of CNS components from the peripheral immune system by tightly regulated barriers, unwanted immune responses and autoimmunity are minimized. However, ischemic stroke induces an evident inflammatory response, characterized by the rapid activation of resident microglia and subsequent infiltration of peripheral leukocytes (11). Various mechanisms, ranging from the secretion of soluble mediators to direct interaction with CNS resident cells, are deployed by immune cells to promote an inflammatory environment within the ischemic brain, inducing cell death and worsening stroke outcome (11, 12). Therefore, alleviating post-stroke inflammation could benefit patients with stroke.

Among all immune components, T cells are especially of interest given their potency in both innate and adaptive immune responses. Several subsets of T cells with various functions exist, but all are types of lymphocytes marked by the expression of CD3 (13, 14). T cells can be divided into the αβ subset, whose T cell receptors (TCR) are heterodimers of the α and β chains, and the unconventional γδ subset, whose TCRs are heterodimers of the γ and δ chains. The αβ subset can be further divided into CD4+ T helper cells (Th), which regulate of the functions of phagocytes, granulocytes, and other lymphocyte subsets, CD8+ cytotoxic T lymphocytes (CTL), which exert a direct cytotoxic role, and regulatory T cells (Treg) that regulate immune responses (14). The surface makers, transcription factors important for differentiation, cytokine secretion, and functions of the major T cell subsets are listed in **Table 1**.

**Table 1:** Major T cell subsets involved in the post-stroke immune response.

Subset	Surface marker	Transcription factor	Cytokine secretion	Function
**αβ T lymphocytes**				
T Helper				
-Th1	CD3+ CD4+, CD8-	T-bet	IFN-γ, TNF-α, IL-2	Defense against intracellular pathogens, macrophage activation and inflammation
-Th2	CD3+ CD4+, CD8-	GATA3	IL-4, IL-5, IL-10, IL-23, IL-33	Defense against parasite infection, B cell activation and allergy
-Th17	CD3+ CD4+, CD8-	RORγt	IL-17, IL-6, IL-21, IL-22	Defense against bacteria and fungi and autoimmunity
Regulatory T cell (Treg)	CD3+ CD4+ CD25+ FOXP3+^*^	FOXP3	IL-10, TGF-β, IL-35	Regulation of immune response and protection from autoimmunity
Cytotoxic T cell (CTL)	CD3+ CD8+, CD4-	–	–	Cytotoxic effects, including defense against virus, bacteria, parasites and tumors. Contribute to some autoimmune diseases.
**γδ T lymphocytes**				
γδ T lymphocytes	γδ TCR	–	–	Pathogen clearance, inflammation and tissue homeostasis

^*^This is the most commonly discussed regulatory T cells and is referred to as Treg throughout this review, although other subsets of Tregs also exist.

The effects of T cells on stroke outcomes have been robustly confirmed (15). Compared with wild-type mice, recombinant activating gene 1 (*Rag*1) -/- mice, which are deficient in T and B cells, develop significantly smaller infarcts areas, demonstrating the deleterious effects of T cells (16, 17). However, it should not be overlooked that distinct mechanisms underlie post-stroke inflammation in different subsets of T cells. Certain subsets of T cells, including Tregs, have shown protective effects (18). Therefore, elucidating the T cell response after ischemic stroke could promote the development of novel stroke therapies.

This review aims to summarize the process of T cell response after ischemic stroke, beginning with describing how and when the T cell response is initiated. Next, we discuss the routes and drivers of T cell infiltration, as well as the mechanisms underlying the deleterious and protective T cell-mediated effects on the ischemic brain. Finally, we focus on experimental studies and clinical trials on potential T cell-targeted therapies.

## T Cell Recruitment and Infiltration to the CNS

Ischemic stroke is caused by a sudden disruption in blood flow resulting from parenchymal vasculature occlusion. Local low-perfusion and hypoxic environments quickly induce metabolic dysfunction and cell death, followed by the release of damage-associated molecular patterns, reactive oxygen species, and ATPs, which all interact with microglia, the brain resident immune cells. Subsequently, microglia are activated, inducing their secretion of cytokines and potentiating blood-derived leukocyte infiltration, and therefore initiate an inflammatory cascade (11).

### Adhesion and Infiltration of T Cells

**Figure 1:**
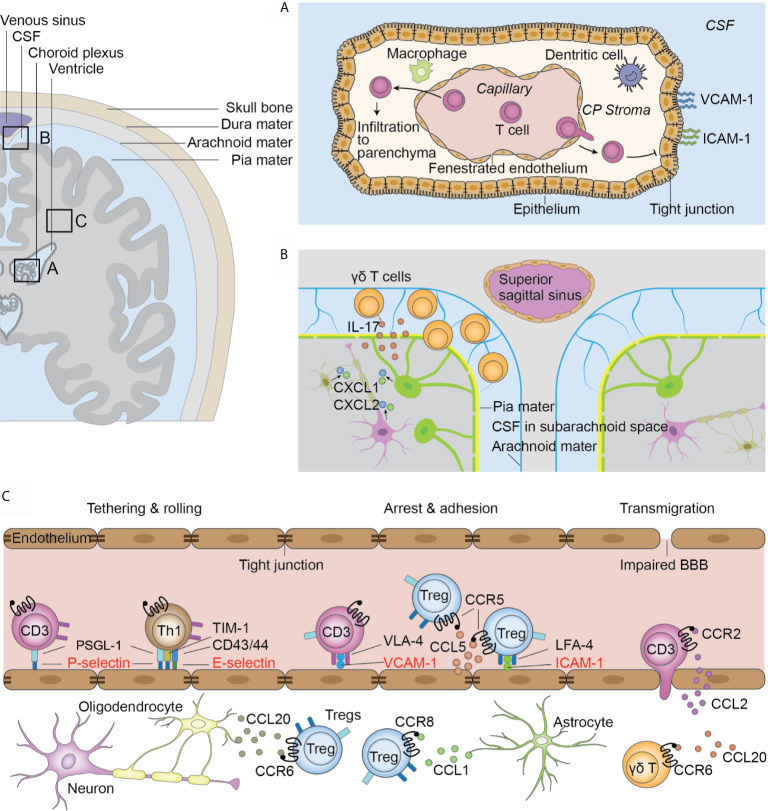
Infiltration routes of T cells after ischemic stroke. **(A)** Choroid plexus (CP) T cells infiltrate to the CP stroma through the fenestrated capillaries. The tight junctions between epithelial cells and lack of adhesion molecules on the stromal side of the CP hinder T cell transmigration to the CSF. T cells in the stroma directly migrate to the parenchyma from the root of CP. **(B)** Meninges. γδ T cells migrate from the intestines to the brain after ischemic stroke and are preferentially located in the leptomeninges. γδ T cells secrete IL-17 and induce the upregulation of CXCL1 and CXCL2 in the parenchyma. **(C)** Impaired blood-brain barrier in the parenchymal vasculature. T cells are initially tethered by the interaction between PSLG-1 and P-selectins on endothelial cells. Th1 cells also use TIM-1-P-selectin and CD43/44-E-selectin interactions for their tethering and rolling. Further adhesion and arrest of T cells are mediated by integrin and their receptors. VLA-4 and VCAM-1 are universally used by all T cell subsets while the LFA-4 and ICAM-1 pair is preferentially used by Tregs. Further, the chemokine and receptor pair CCL5 and CCR5 facilitates Treg docking on endothelial cells. Chemokine and receptor interaction facilitates interstitial locating in a cell-type-specific fashion.

T cell trafficking marks the beginning of the T cell response in ischemic stroke. Leucocytes flow at high speeds in blood vessels in homeostatic conditions. However, in ischemic stroke, following phases of tethering, rolling, arrest, and adhesion, T cells attach to the endothelium through the combination specific adhesion molecules (19). The initial tethering and rolling of T cells is mediated by the binding between endothelial selectins and its T cell ligands (20). Selectins are a family of three closely related glycoproteins, including P-selectin expressed on platelets and the endothelium, E-selectins expressed on vascular endothelium, and L-selectin expressed on leukocytes (21). In ischemic stroke, the level of P-selectin and E-selectin on endothelial cells are upregulated in response to the cytokines secreted by microglia (22). The predominant ligand of P-selectin, P-selectin glycoprotein ligand 1 (PSGL-1), is constitutively expressed by all subsets of T cells (23). In contrast, T cell immunoglobulin and mucin domain 1 (TIM-1), another P-selectin ligand, cooperates with PSLG-1 to mediate the tethering and rolling of Th1 and Th17 cells, but not Th2 and Tregs in the inflamed CNS microvasculature (24). The E-selectin ligands expressed on T cells include CD43 and CD44, and facilitate Th1 cell tethering in inflammation (21, 25). Whether these E-selectin ligands are also utilized by other T cell subsets remains unclear (**Figure 1C**).

The initial tethering and rolling of T cells increase the chance of interaction between T cell integrins and their endothelial Ig superfamily ligands, which are necessary for their subsequent firm arrest on the endothelium (26–28). VLA-4 and VCAM-1 is a well-characterized integrin-ligand pair, with VLA-4 being widely expressed on peripheral blood lymphocytes and VCAM-1 expressed in endothelial cells at an upregulated level in ischemic stroke (29). This integrin pair is universally used by all T cell subsets, as blocking VLA-4 with an antibody of its α4-integrin unit reduced the infiltration of Th, CTL, and Treg cells to the brain and reduced the infarct volume (30–32). Other integrin pairs, however, are preferentially used by specific T cells subsets. Tregs preferentially use the adhesion molecule pair LFA-1 and ICAM-1, and express the highest levels of LFA-1 among all T cell subsets (33). *In vitro* adhesion assays revealed a more prominent binding capacity of Tregs to ICAM-1 compared with other CD4+ T cells (33). Similarly, blocking LFA-1 exerted protective effects on *Rag*-/- mice that underwent adoptive transfer of CD4+ CD25+ Tregs, but not other CD4+ cells (**Figure 1C**) (33). Whether other T cell subsets use specific integrin pairs remains to be explored.

Chemokines, a large family of cytokines with chemotactic activity, mediate intravascular lymphocyte adhesion, as well as their interstitial migration and positioning (28, 34). T cells express various chemokine receptors. *Ccr2*-/- mice show less T cell infiltration into the parenchyma, and there is an upregulation of CCL2 in the cortex after ischemic injury, suggesting the crucial role of CCL2/CCR2 interaction in over-all T cell infiltration (35). The chemokine receptor expression profile in T cells shifts with the temporal course of stroke. As early as 24 hours after middle cerebral artery occlusion (MCAO), a mouse model of ischemic stroke, there is an upregulation of CCR5 in CD4+ CD25+ Tregs. Together with the upregulation of its ligand CCL5 in endothelial cells at the injured site, this CCL5/CCR5 interplay mediates Treg docking to the injured endothelium and prolongs the time of contact between Tregs and endothelial cells, and therefore help to maintain Tregs at the lesion site (36). At subsequent stages of ischemic stroke (day 14), Tregs shift to increased expression of CCR8 and CCR6. An upregulated expression of their ligands, CCL1 and CCL20, in astrocytes and oligodendrocytes in the parenchyma, respectively, are also observed (37). Different chemokine receptors are preferentially expressed on certain T cell subsets, which mediates cell type-specific infiltration. For example, brain-infiltrating IL-17 producing γδ T cells naturally express CCR6. CCR6 deficiency in these cells decreases their post-MCAO infiltration while the number of CD4+ and CD8+ T cells remain unaltered, suggesting the preferential use of CCR6 in γδ T cell migration (38). The preferential use of chemokine signaling by other T cell subsets remains to be explored. Taken together, these findings indicate that the temporal-dependent and cell-type-selective chemokine/receptor interaction direct the post-stroke “fine-tuning” of T cell infiltration in the brain (**Figure 1C**).

### Routes of T Cell Infiltration

In ischemic stroke, three routes of T cell infiltration are proposed, i.e., the blood-brain barrier (BBB) (**Figure 1C**), choroid plexus, and meninges (39). In a mouse model of transient MCAO, a significant dysfunction of blood brain barrier (BBB) occurs 2 h after reperfusion in the distal capillary and venular microvascular beds (40). Further, this dysfunction is observed in human patients as early as at 3 hours after stroke, as demonstrated by contrast enhancement on T1-weighted imaging (41). The mechanism of BBB dysfunction and its inflammatory response have been comprehensively reviewed elsewhere (42, 43). The impaired BBB integrity gives way for T cell infiltration (42, 44).

Another significant pathway for T cell infiltration is the choroid plexus (**Figure 1A**). The choroid plexus is a plexus of cells located in the lateral, third, and fourth ventricles with the major function of cerebrospinal fluid (CSF) production (45). It comprises of the innermost layer of fenestrated capillaries surrounded by connective tissues, which are termed the stroma, and the outermost layer of epithelial cells connected by tight junctions that is continuous with ependymal cells that lines the ventricles (39). Together, these structures form the blood cerebral spinal fluid barrier (BCSFB). The fenestrated endothelial cells facilitate immune cell infiltration into the stroma, while the tight junctions between epithelial cells and the exclusive expression of adhesion molecules VCAM and ICAM on the ventricular side hinder immune cell migration to the CSF (46). Fluorescent tracing show that approximately two-thirds of all infiltrated T lymphocytes in the ischemic parenchyma originate from the choroid plexus of the ipsilateral lateral ventricle (35). Only choroid stroma infarction, but not CSF circulation blockage, could reduce the number of infiltrated T lymphocytes, suggesting that T cells directly enter the parenchyma through the stroma of the choroid plexus, rather than by passing the tight blood-CSF barrier and then entering the parenchyma (35). Nevertheless, activated T cells can be found in the CSF of human patients (47). Further studies are thus required to confirm the infiltration route of T cells from the choroid plexus to the parenchyma.

Additionally, T cells migrate from the meninges to the brain (**Figure 1B**). In patients with ischemic stroke, the accumulation of T cells in the meninges can be observed within the first 3 days post insult (47). Using fluorescent cell tracing, Benakis et al. reported mobilization of γδ T cells from the intestines and specific accumulation in the leptomeninges early after MCAO (48), accompanied by increased levels of IL-17 and the chemokines CXCL1 and CXCL2 in the meninges (48). However, it remains unclear whether these cells proceed to infiltrate the parenchyma. Meningeal γδ T cells have been shown to mediate anxiety-like behaviors in mice through neuronal IL-17Ra signaling under homeostatic conditions (49). Future studies should examine whether the increased post-stroke accumulation of translocated γδ T cells mediates post-stroke behavioral changes.

### Temporal Course of T Cell Infiltration

CD3+ T cell infiltration is evident as early as the first 24 hours after MCAO in animal models (50). In transient MCAO models, the peak of T cell infiltration appears at 3–5 days after stroke induction (50–53). In permanent MCAO (pMCAO) models, T cells infiltration peak at a relatively delayed timing of 7 days after stroke, although a solitary study reported an early peak at 6 h (54). Although most studies focused on the first 7 days after ischemic stroke, it should be noted that T cell accumulation in the brain continue to happen after 7 days. A long-term T cell response, represented by the presence of T cells in the parenchyma and their expression of active-state markers, lasts until at least day 28 (55, 56). Different T cell subsets do not act as synchronized troops during infiltration into the brain. **Table 2** presents the published data on the temporal course of infiltration of T cell and its subsets.

**Table 2:** Temporal course of T cell infiltration to the brain parenchyma.

Strain	Model	Cell type	Method	Observation	Ref
C57BL/6J	PO	CD3+ T cells	IHC	T cell count keeps rising from day 1 to day 28.	(55)
				T cells accumulate in the infarct core and corpus callosum.	
C57BL/6J	PO	CD3+ T cells	M	T cell count peaks at 7 days and begins to decrease till day 21.	(57)
CB-17 mice	PO	CD3+ T cells	IHC	T cell count peaks at 6 hours after stroke and is decreased at 24h. The count slightly rises from day 1 to day 7.	(54)
C57BL/6	PO	CD3+ T cells	IHC	T cell count significantly increases from day 1 to day 5.	(30)
				T cells accumulate in the peri-infarct zone.	
Sprague‐Dawley rats	PO	CD3+ T cells	IHC	T cell count rises from 6h to 6 days after stroke.	(58)
C57BL/6	60 min TA	CD3+ T cells	IHC, F	T cell count increases from day 1 to day 7 after ischemic stroke and peaks at 3 days.	(52)
C57BL/6	60 min TA	CD3+ T cells	F	T cell count increases from day 1 to day 6 after ischemic stroke, with a peak on day 3.	(53)
C57BL/6	60 min TA	CD3+ T cells	F	T cell count increases from day 1 to day 7 and peaks on day 3.	(50)
C57BL/6J	30 min TA	CD4+ T cells	FACS	CD4+ T cell count continues to rise from day 7 to day 30.	(59)
C57BL/6	60 min TA	CD4+ T cells	F	CD4+ T cell count slightly increases from day 1 to day 6 after stroke.	(53)
C57BL/6J	60 min TA	CD8+ T cells	F	CD8+ T cell count increases from day 1 to day 3 after ischemic stroke.	(60)
C57BL/6J	30 min TA	Tregs	FACS	Tregs cell count rises from day 7 to day 30, and is significantly higher than day 7.	(59)
				Tregs are located in the peri-infarct area, infarct area, had trespassed brain vessels on day 14.	
C57BL/6J	60 min TA	Treg	F	Tregs can be observed on day 3 and 5 after ischemic stroke. Numbers significantly increase on day 7 and escalate until at least day 35.	(61)
C57BL/6	60 min TA	γδT cells	F	γδT cell count increases from day 1 to day 6 after ischemic stroke and peaks on day 3.	(53)
				γδT cells are located in infarct boundary zones.	
C57BL/6J	60 min TA	CD4- CD8- T cells	F	CD4- CD8- T cell count increases from day 1 to day 3 after ischemic stroke.	(60)

PO, permanent occlusion; TO, transient occlusion; F, flow cytometry; FACS, fluorescence-activated cell sorting; IHC, immunohistochemistry; M, magnetic cell sorting.

In patients with ischemic stroke, T cell numbers have been shown to increase from day 1 to at least day 124 after stroke, with an escalated speed of accumulation between days 8 and 20 (47, 62). An increased count of lymphocytes and CXCL-11, a T cell chemoattractant, in arterial blood drawn from the distal side of the occlusion site during mechanical thrombectomy, can already be found (63), suggesting that T cell recruitment in humans begins in the hyperacute phase of ischemic stroke. In addition, robust infiltration of activated T cells into the infarct brain has been reported on day 140 day after stroke, of which > 60% are CD3+ CD8+ T cells, suggesting a long-lasting T cell response in the human ischemic brain (47). There is, however, a lack of information on the temporal infiltration of different subsets of T cells in human patients.

## Antigen-Independent and Antigen-Dependent T Cell Response

T cells are involved in post-stroke inflammation in both antigen-dependent and antigen-independent manners. In the early phase of ischemic stroke, T cells react in an antigen-independent manner and are closely associated with infarct volume development. This conclusion is inferred based on the following findings. First, an antigen-dependent response requires TCR binding with its specific antigen processed and presented by antigen-presenting cells (APC), as well as signals from costimulatory molecules (64). However, on day 1 after ischemic stroke, transgenic mice with CD4+ or CD8+ T cells bearing a uniform TCR, mice deficient in costimulatory molecules CD28, B7-H1, or PD1 are as fully susceptible to ischemic reperfusion injury as their wild-type littermates (16). This suggests that the deleterious effect of T cells occurs in the absence of antigen recognition. Second, certain T cell subtypes, including γδ T cells, naturally do not require antigen recognition for activation (38, 65). Depletion of γδ T cells has been shown to exert protective effects only after 3 days post stroke, a time when their IL-17 secretion became evident (53). Third, by measuring TCR diversity of specific TCR hypervariable region genes, Liesz et al. confirmed that clonal expansion of T cells, the hallmark of T cell antigen recognition, first appeared on 7 days after MCAO (57). This is consistent with the fact that antigen recognition usually takes 3 to 7 days (64). The temporal dissociation between adaptive immune response and early T cell-dependent effects indicate that the early deleterious effects of T cells, at least within the first 3 days, are not antigen-dependent. **Table 3** summarizes the antigen-dependent and -independent responses of T cells.

**Table 3:** Antigen-dependent and antigen-independent responses of T cells in ischemic stroke.

Subset	Mechanism	Target cell	Effect	Time	Ref
**Antigen-dependent responses of T cells**					
CD8+ T cells	Direct cytotoxicity by Granzyme B, perforin	Neuron	Neuronal apoptosis; increased infarct volume	7,14 days	(30, 66)
Treg	Secretion of amphiregulin	Astrocyte	Inhibition of astrogliosis	14,30 days	(37)
**Antigen-independent responses of T cells**					
CD3+ T cells	Secretion of IFNγ	Macrophage Th1 cell	Facilitating TNFα secretion from macrophage, urging infiltration of Th1 cells	3 days	(50, 67)
CD4+ T cells	Secretion of IL-21	Neuron	Neuron autophagy, increased infarct volume?	24 hours	(68, 69)
γδ T cells	Secretion of IL-17	Neutrophil T cell	Facilitating neutrophil and T cell infiltration; increased infarct volume	3 days	(53)
CD3+ CD4-CD8- T cells	Secretion of TNFα	Microglia	Promoting microglia phenotype change to the inflammatory type	1 to 3 days	(60)
Treg	Interaction with platelets	–	Microvascular dysfunction, micro-thrombosis	24 hours	(33)
CD3+ T cell	Binding with CD84 from T cells and platelets	Platelet	Facilitating T cell infiltration; increased infarct volume	24 hours	(70)
Treg	Secretion of IL-10	Microglia	Inhibition of IFNγ and TNFα secretion from microglia; decreased infarct volume	24 hours	(18)
Treg	Secretion of IL-10	Neuro stem cell	Boosting neuro stem cell proliferation	4 days	(71)
Treg	Interact with PD-1 on neutrophils	Neutrophil	Inhibiting production of MMP-9; protecting BBB integrity	3 days	(72)
Treg	Secretion of osteopontin	Microglia	Phenotypic change to reparative microglia, boosting oligodendrocyte differentiation	21 days	(61)

Antigen-dependent T cell responses occur in the later stages of ischemic stroke. Adoptive transfer of CD8+ cytotoxic T lymphocytes (CTL) obtained from OVA-specific ovalbumin transgenic mice, which only have TCRs specific to a chicken protein, to *Rag*-/- mice caused a smaller infarct volume at 7 days and 14 days after stroke and a reduced number of infiltrated CTLs in the parenchyma (73). Additionally, there was no infiltration of CD4+ T cells in *Rag*-/- OTII transgenic mice on day 14 after ischemic stroke (37). Furthermore, Treg development in the brain is antigen-dependent since Tregs infiltrating the brain on day 14 have a much less diverse TCR repertoire compared with splenetic Tregs, and the most abundant TCR of brain-infiltrated Tregs accounts for > 5% of the entire TCR repertoire (37). Taken together, these findings suggest that in the later stages of ischemic stroke, there is a gradual development of antigen-dependent T cell responses.

**Figure 2:**
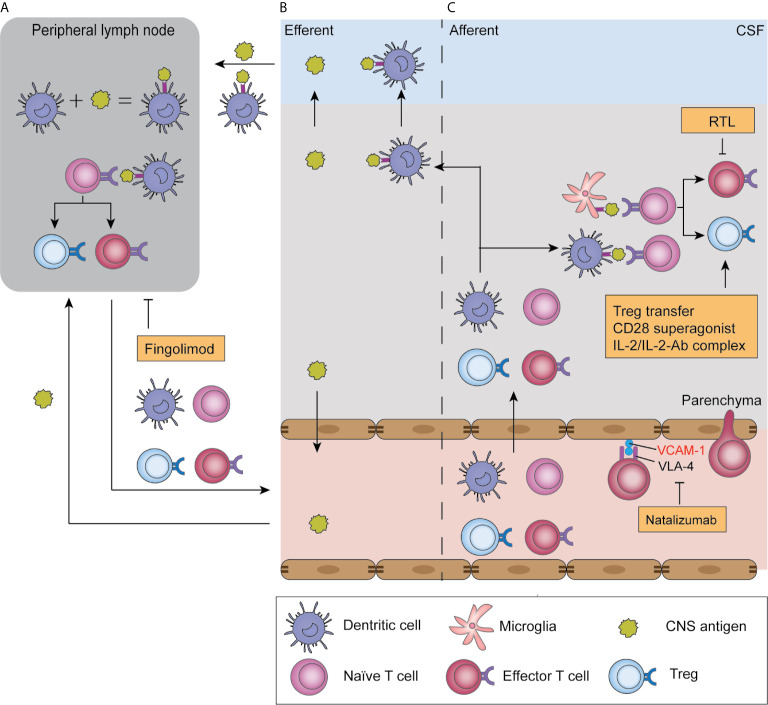
Antigen presentation in ischemic stroke and T cell-related therapeutic targets. After ischemic stroke, soluble antigens are leaked to the blood or CSF and reach draining lymph nodes; alternatively, they are presented by infiltrated dendritic cells and migrate with these cells **(B)**. In peripheral lymph nodes, antigens are presented by antigen-presenting cells and are recognized by naïve T cells, which triggers their proliferation and production of effector T cells and Tregs, a process termed “T cell priming” **(A)**. Moreover, T cell priming also occurs in the parenchyma, where infiltrated naïve T cells encounter antigens presented by microglia and dendritic cells. Subsequently, primed T cells migrate to the parenchyma where they reencounter their specific antigen and exert their function in an antigen-dependent way **(C)**. T-cell-targeted therapies include natalizumab, which blocks T cell adhesion to endothelial cells by blocking the adhesion molecule VLA-4. Fingolimod blocks T cell egression from lymph nodes, and therefore induces lymphopenia. Recombinant T cell ligands (RTL) block effector T cell functions, while Treg-targeted therapies boost Treg function.

The development of a classic antigen-dependent T cell response includes two steps: priming and reactivation. T cell priming refers to the activation of naïve T cells to proliferate and produce effector cells during their initial encounter with their specific antigens. To fully execute their function, T cells migrate to the site of their specific antigen and are reactivated by antigens on target cells or local APCs. Canonically, T cell priming occurs in the peripheral lymph nodes (**Figure 2A**), which is also feasible in ischemic stroke given that impaired barrier function allows leakage of soluble antigens from the BBB to blood or from interstitial extracellular fluid to perivascular spaces around arterioles then to the CSF, and subsequently reach the draining lymph nodes where they are processed by resident DCs (**Figure 2B**) (74, 75). Notably, infiltrated DCs and microglia in the brain are also capable of antigen presentation and subsequent migration to the cervical lymph nodes (**Figure 2B**) (76). Indeed, there is an increase in neural and myelin antigens, including MAP-2, MBP, and MOG, in macrophages, as well as migrated DCs, in the cervical lymph nodes of patients with stroke (77). However, recent findings have challenged the idea of T cell priming solely occurring in peripheral lymph nodes. Clonal expansion of T cells can be detected in the brain on day 7 after stroke and is delayed until day 14 in the spleen and lymph nodes (37, 57, 78). This temporal lead of T cell priming in the brain suggests that in the early phase of ischemic stroke, naïve T cells migrate to and are primed in the brain by local microglia or infiltrated DC cells (**Figure 2C**). Taken together, these findings suggest that early T cell priming primarily occurs in the brain and peripheral activation occurs at later time points.

Dural sinuses have recently been identified as a neuroimmune interface. The dural sinuses are venous channels found between the endosteal and meningeal layers of the dura mater. Dural sinuses harbor populations of resident APCs and patrolling T cells, enabling it for initiating a T cell response. In a mouse model of EAE, soluble antigens in the CSF efflux to the peri-sinusal dura, are captured by dural APCs, and presented to patrolling T cells (79). This route remains to be researched in the context of ischemic stroke.

Antigen-specific T responses could be both deleterious and beneficial. Interestingly, in human patients, increased reactivity to neural antigens is associated with smaller infarctions and better outcomes, while reactivity to myelin antigens is correlated with worse outcomes (77). The nature of an antigen-specific response is highly relevant to the local environment, with DC cells being crucially involved in pivoting this process. Mature DCs induce immunity while immature DCs induce tolerance through anergy induction or conversion of naïve T cells to Tregs (80). Induction of immunotolerance by repetitive low-dose feeding of stroke-related antigen prior to stroke induction improves stroke outcome. Oral tolerance to myeline basic protein decreases the infarct size at both 24h and 96h after MCAO (81), witch triggers Treg formation and secretion of anti-inflammatory cytokines IL-10 and TGF-β (82). Mucosal tolerance to E-selectin, an adhesion molecule expressed by endothelial cells under inflammatory conditions, is protective in ischemic stroke, which reduces the number of infiltrated T cells and the infarction size (83). Therefore, manipulating antigen-specific responses could be a potential therapeutic target.

## Mechanism of T Cell-Mediated Effects

### Detrimental Effects of T Cells

**Figure 3:**
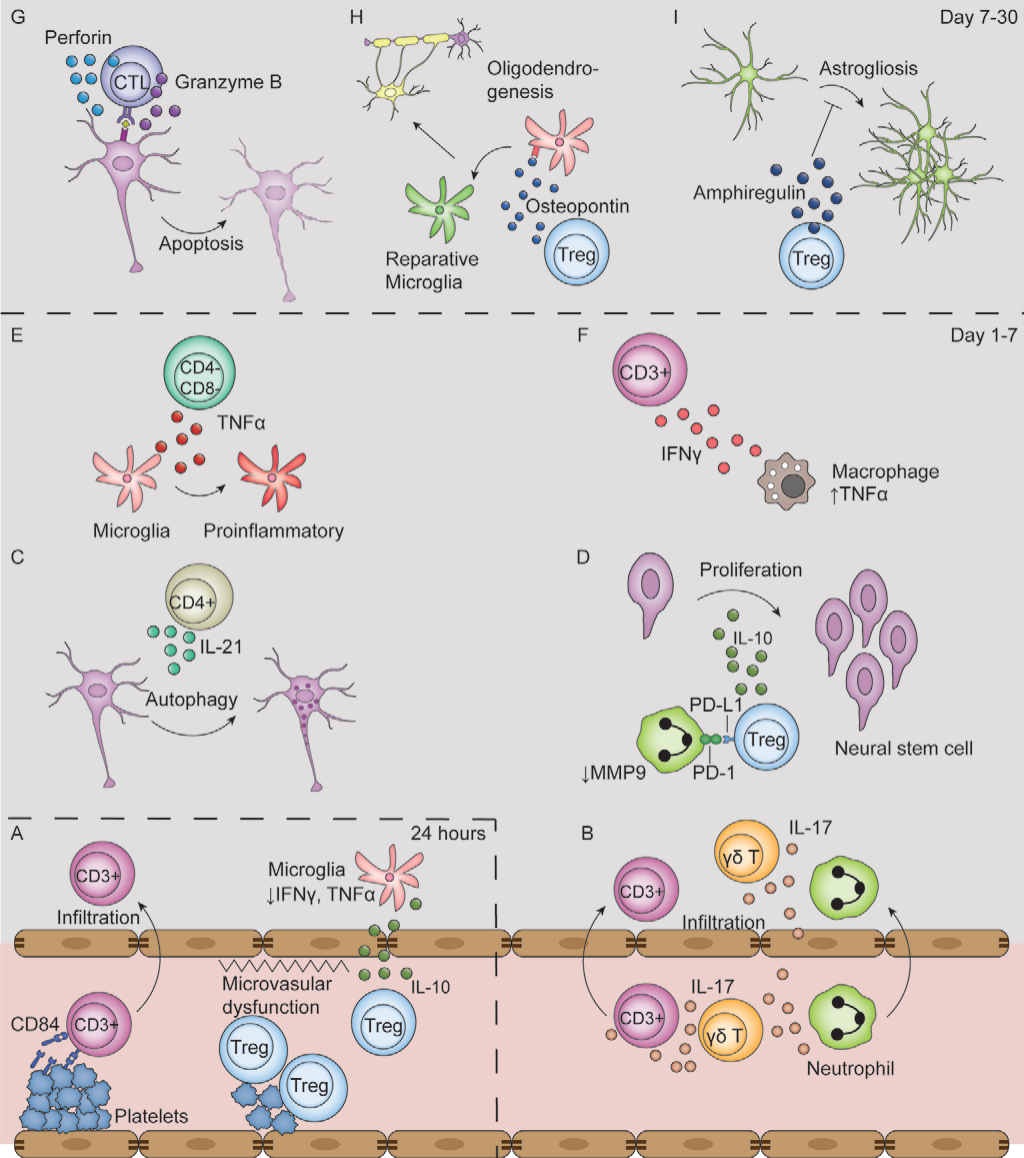
T-cell mediated effects in the ischemic stroke brain. **(A)** In the first 24 hours after ischemic stroke, Tregs interact with platelets in the vasculature, which induces thrombosis and microvasculature dysfunction. Moreover, Tregs secrete IL-10 to decrease microglial secretion of IFN-γ and TNF-α in the absence of their infiltration to the parenchyma. Additionally, T cells bind with CD84 shed from platelets and promote their own infiltration. **(B–F)** From day 1 to 7 after ischemic stroke, T cells secrete various types of cytokines, including IL-17 **(B)**, IL-21 **(C)**, IL-10 **(D)**, TNF-α **(E)**, IFN-γ **(F)**. Moreover, Tregs decrease MMP9 production by neutrophils through PD-L1 and PD-1 interaction **(D)**. **(G–I)** From post-stroke day 7 to 30, CTLs induce neuronal apoptosis by perforin and granzyme B secretion in an antigen-dependent way **(G)**. In addition, Tregs secrete osteopontin to induce a reparative microglia phenotype and therefore promote oligodendrogenesis **(H)**, and secrete amphiregulin to inhibit deleterious astrogliosis **(I)**.

In the challenge of ischemic stroke injury, *Rag1*-/- mice develop significantly smaller infarcts after MCAO (16, 17). Neither B cell deficiency nor its reconstitution in *Rag1*-/- mice affects the infarct volume, while adoptive transfer of T cells to *Rag*-/- mice significantly increased infarct volume. These findings confirm the dominant role of T cells in the deleterious effects of lymphocytes after ischemic stroke (17, 84). These T cell effects can be observed as early as 24 h after ischemic stroke, and T cells in the brain remain activated for at least 30 days (56). Depletion of either CD4+ or CD8+ T cell subsets reduced infarct volume at 24 hours after ischemic stroke (30, 53); moreover, depletion of γδ T cells decreased the infarct volume on day 4. Collectively, these findings are indicative of the detrimental role of these T cell subsets. The mechanisms of T cell-mediated effects are presented in **Figure 3**.

#### Production of Inflammatory Cytokines

##### IFN-γ

T cells are strongly associated with the activation and cytokine secretion by macrophages through IFN-γ production. *Rag*-/- mice showed reduced levels of TNF-α produced by macrophages (**Figure 3F**); additionally, the reconstitution of T cells from *Ifng-/-* mice could not render the TNF-a level to that in mouse reconstituted with wildtype T cells, indicating that IFN-γ produced by T cells facilitates TNF-α production by infiltrated macrophages (50). Furthermore, IFN-γ acts on T cells themselves. It induces the production of interferon-gamma-inducible protein (IP-10), also known as CXCL10, whose ligand CXCR3 is selectively expressed on Th1, but not Th2, cells, and therefore selectively urges Th1 cell infiltration (67). Blocking IFN-γ with an antibody or its genetic deletion decreased number of infiltrated T lymphocytes and decreased infarction volume (17, 85).

##### IL-21

The level of IL-21 in the brain is increased after ischemic stroke with infiltrated CD4+ T cells being its major source (68). IL-21 interacts with the IL-21 receptor on neurons and induces autophagy *in vitro* (**Figure 3C**) (68). *Rag*-/- mice receiving CD4+ T cells from *Il-21*-/- mice developed significantly smaller infarct volumes compared with those receiving wildtype CD4+ T cells at 24 h after MCAO (68). Regardless, contradictory results are found as mice deficient in IL-21 receptor developed larger infarct volumes and exacerbated neuron loss through apoptosis (69). In human patients, IL-21 colocalizes with CD4 in postmortem brain tissues, indicative of its potential for clinical translation (68).

##### IL-17

IL-17 producing γδT cells, but not Th17 cells are the major source of IL-17 in the acute phase of ischemic stroke (53). γδT cells are a subset of unconventional T cells whose TCRs comprise the Vγ and Vδ chains. Compared with conventional αβ T cells, γδT cells do not recognize MHC-associated peptide antigens and are not MHC restricted. The secretion of IL-17 by γδT cells is not evident until day 3 after ischemic stroke, in line with the fact that the depletion of γδT cells only showed protective effects after day 3 (53). The secretion of IL-17 from γδT cells is dependent on IL-23, the major source of which is infiltrated DCs (86). IL-17 production is significantly reduced in *Il-23*-/- mice (53), and IL-23 p19 knockdown suppressed IL-17 mRNA and protein levels (87).

IL-17 acts on various cell types in the CNS, including astrocytes, neurons, and microglia. IL-17, in synergy with macrophage-produced TNF-α, mediates the secretion of CXCL-1, a neutrophil chemoattractant, by astrocytes (**Figure 3B**) (50). Administration of an IL-17 blocking antibody 3 hours after stroke significantly reduced neutrophil infiltration, infarct volume, and improved neurological outcome 3 days after MCAO (50). IL-17 also stimulates the IL-6 signaling pathway in astrocytes and acts in synergy with IL-6 to increase CCL20 levels in astrocytes, which facilitates T cell migration *in vitro* (88). Moreover, IL-17 directly acts on neurons in a dose-dependent manner to impair neural viability *in vitro* through its interaction with IL-17R (89). Additionally, IL-17 promotes the expression of IL-6, macrophage inflammatory protein-2, nitric oxide, adhesion molecules, and neurotrophic factors by microglia *in vitro* (90).

##### TNF-α

Although macrophages are the major TNF-α source after ischemic stroke, CD3+CD4-CD8- T cells also secrete TNF-α on day 1 to day 3 and is located around the Iba1+ microglia (60). TNF-α acts on microglia to promote a proinflammatory phenotype change, and therefore exacerbates post-stroke inflammation (**Figure 3E**) (60).

#### *Thrombo*-Inflammation

The term “thrombo-inflammation” refers to the exacerbated infarct development after ischemic stroke mediated by T lymphocyte-platelet interactions (91). This effect is already evident in the hyperacute phase of ischemic stroke and is a major factor in the tissue loss observed in the penumbra (92). Blocking platelet glycoprotein receptor Ib (GPIb), an adhesion receptor crucial for platelet binding to the endothelium during thrombosis, can specially reduce infarction volume in the border of the penumbra immediately after 2 hours of MCAO (92). This protective effect of platelet blockage can be observed even 24 hours after reperfusion (93, 94).

Thrombo-inflammation is a combined result of both microvascular dysfunction and exacerbated inflammation (**Figure 3A**). Adoptive transfer of Tregs to *Rag*-/- mice resulted in an increased infarct size dependent on the presence of platelets (33). The presence of both Tregs and platelets reduced cerebral blood flow and increased brain fibrin levels compared with platelet-depleted mice while the Treg immunological function remains unchanged, indicative of platelet-Treg-associated microvascular dysfunction and thrombosis (33). Evidence for microvascular dysfunction induced by other subsets of T cells remain to be found. Moreover, platelets facilitate T cell infiltration into the brain parenchyma. Blocking either GPIb or its ligand VWF A1 domain with an antibody, or their genetic deletion, significantly reduced the number of infiltrated T cells and infarct volume (92–94). Another platelet-born pro-infiltration factor is CD84, a soluble homophilic immunoreceptor that is shed from platelets after ischemic stroke (70). It binds to CD84 on T cells and enhances CD4+ lymphocyte migration *in vitro* (70). Mice deficient in platelet or T cell CD84 developed smaller infarct volumes and had fewer infiltrated T cells (70). Notably, higher CD84 levels on platelets were associated with worsened clinical outcomes in human patients (70).

Taken together, these findings suggest that T cell and platelet interactions exacerbate ischemic injury through thrombo-inflammation. However, there remains insufficient evidence regarding the direct binding of T lymphocytes and platelets and future studies are needed.

#### Direct Cytotoxicity

CD8+ T cells, also known as CTLs, are crucially involved in the adaptive immune response. These cells exert a cytotoxic function by antigen-recognition from the TCR and the subsequent release of granzymes and perforin, forming pores on target cells and inducing apoptosis (95, 96). The deleterious effects of CD8+ T cells predominantly occurs in the chronic phase due to its adaptive nature. Higher numbers of ipsilateral CD8+ cells on day 30 after MCAO is correlated with worse functional outcomes in mice (97). Delayed depletion of CTLs with a CD8 antibody from day 10 after ischemic stroke improved functional recovery in mice (97).

CTL depletion in mice significantly reduced infarct volume and improved neurological functions at days 7 and 14 after ischemic stroke and CTL reconstitution to *Rag*-/- mice increased infarct volume (73). The direct cytotoxic effects of CTLs are dependent on their release of granzyme B and perforin. The protein level of Granzyme B is elevated in post-stroke human samples (66). Immunofluorescence studies reported granzyme B co-localization with CD8+ T cells and terminal deoxyuridine nick-end labeling-positive neurons, indicative of granzyme B-mediated neuronal apoptosis (66, 98). Moreover, perforin is released by CTLs to induce neuronal damage. CTL reconstitution from *Prf-/-* mice to *Rag*-/- mice exerted protective effects compared with reconstitution of CTLs from wildtype mice, indicative of a perforin-dependent cytotoxic effect (73). The combined use of a VLA-4 antibody, which blocks T cell infiltration into the brain parenchyma, with *Prf-/-* CTL reconstitution did not have an additive effect, suggesting that the perforin-dependent killing effect is dependent on the presence of T cells in the parenchyma rather than collateral effects on the luminal side (30). Reconstitution of CTLs genetically edited to have a TCR exclusively reactive to chicken ovalbumin did not alter stroke outcome, which suggests an antigen-dependent manner of CTL cytotoxicity (30). Taken together, these findings indicate that infiltrated CTL cause worsened post-stroke outcomes through an antigen-specific and contact-dependent pathway (**Figure 3G**).

### Protective Effects of T Cells

Regulatory T cells (Tregs) are a subset of T cells that comprise 5%–10% of the peripheral CD4+ T cell population and are characterized by their transcription factor FOXP3 (99). Tregs are crucial for maintaining immune homeostasis and inducing immune tolerance; moreover, their dysregulation has been implicated in various autoimmune diseases and chronic inflammation (100). Expectedly, the protective effects of T cells after ischemic stroke are mainly exerted by Treg cells given that Treg depletion resulted in the expansion of lesion volume on day 3 to 7, unregimented neutrophil infiltration, and T cell activation (18).

#### Immunomodulation

The early protective effects of Tregs are independent of their infiltration into the parenchyma, but are rather results of immunomodulation in the circulation. Treg depletion in a mouse model of ischemic stroke showed deleterious effects as early as day 1 after stroke (18), while Treg infiltration was not evident until day 5 (101). This protective effect of Tregs is mainly attributed to its immunomodulatory function through IL-10 secretion (102). Tregs regimented the production of IFN-γ and TNF-α by microglia and other T cells (**Figure 3A**); further, IL-10 substitution in Treg-depleted mice could replicate this protective effect (18). IL-10 supplementation to the brain decreased infarct volume and downregulated the expression of inflammatory genes such as IL-1β (103). In human patients, a lower serum level of IL-10 is associated with neurological functional deterioration within the first 3 days after stroke (104).

Tregs also exert their immunomodulatory effects through cytokine-independent pathways (**Figure 3D**). PD-L1 on Tregs interact with PD-1 on neutrophils to inhibit their MMP-9 production, and therefore protects BBB function (72). This protective effect was lost in mice transferred with Tregs pretreated with PD-L1 antibody or PD-L1 deficient Tregs (72). Tregs also downregulate costimulatory molecules CD80 and CD86 on DCs by binding with cytotoxic T lymphocyte-associated antigen-4, as well as by inducing an immunoreceptor tyrosine-based activation motif-mediated inhibitory signaling pathway through the interaction of lymphocyte activation gene 3 with major histocompatibility complex (MHC) class II molecules. However, these pathways have not been studied in the field of ischemic stroke (100).

#### Neuro-Repair

Treg depletion starting 7 days after ischemic stroke did not alter infarct volume and neuronal tissue loss in the later stages. However, Treg depletion significantly worsened the neurological outcomes, suggesting an important role for Tregs in neuro-repair in the chronic phase of stroke (37, 61). Treg facilitate neuro-repair by inhibiting excessive astrogliosis, promoting oligodendrogenesis and promoting neuro stem cell proliferation. In the pathophysiological condition of ischemic stroke, reactive astrocytes lose their normal neuro-trophic function and become hyperplastic and hypertrophic, resulting in astrogliosis and the formation of glia scars, which hinder neuro-repair (105). On day 14 and 30 after stroke, Tregs are located close to GFAP+ astrocytes (59). The depletion of Tregs increases the number of reactive astrocytes and enhances the expression of neurotoxic genes (37). Mechanically, Tregs express high levels of amphiregulin, an epidermal growth factor receptor ligand that downregulates the STAT3 pathway in astrocytes, to inhibit astrogliosis (**Figure 3I**) (37). This effect is very likely to be antigen-dependent given that infiltrated Tregs have very similar TCRs (37).

Tregs also facilitate white matter injury repair by promoting oligodendrogenesis. On day 35 after ischemic stroke, T cells accumulate in the white matter areas enriched in myelin basic protein. Depletion of Tregs causes a decrease in re-myelination, thinner myelin sheaths, reduced nerve fiber conduction from myelinated axons in the corpus callosum, and reduced numbers of newly-generated oligodendrocytes (61). The secretion of osteopontin by Tregs mediates a tissue-reparative microglial phenotype change, therefore boosting oligodendrocyte precursor cell differentiation and promoting white matter repair (**Figure 3H**) (61).

Tregs also contribute to neuro-repair by interacting with neural stem cells. Injection of activated Tregs, characterized by CD44 and CD62L upregulation, into the ventricle promoted neural stem cell proliferation in the subventricular zone on day 4. This effect was no longer present when Tregs were injected together with an anti-IL-10 antibody (71). Additionally, *in vitro* experiments confirmed that IL-10, but not TGF-β, is the main effector in the proliferation of neural stem cells (**Figure 3D**) (71). Newborn neural stem cells can migrate into the infarct area (106) and form synapses with pre-existing neurons (107), and therefore could potentially promote neurological functional recovery.

## T Cell-Targeted Therapies

### Blockage of T Cell Infiltration

#### Fingolimod

Fingolimod was approved by the FDA for the treatment of relapse-remitting multiple sclerosis (MS) in 2010. Its major mechanism involves acting as a selective sphingosine-1-phosphate 1 antagonist and mediating irreversible receptor internalization in lymphocytes, which is crucial in lymphocyte homing and trafficking (**Figure 2**) (108, 109). Consequently, T cells are sequestered in lymph nodes and peripheral lymphopenia is induced (110, 111). Fingolimod also acts as an unselective agonist on S1P2, S1P3, S1P4, and S1P5 receptors, which are expressed on microglia, astrocytes, oligodendrocytes, and neurons (108). Fingolimod downregulates ICAM expression on endothelial cells, and therefore reduces leukocyte adhesion to vessel walls (112). Other CNS effects of fingolimod, including reduced neuronal apoptosis (112–115), protection of BBB integrity (110, 115), and local cytokine levels (114) are controversially reported.

Fingolimod administration immediately after reperfusion decreased infarct volume at day 1 and day 3 in murine model of transient stroke (113, 115–117). Repeated fingolimod administration in the first three days after stroke showed a protective effect lasting for up to 15 days (112). Additionally, fingolimod reduced infarct volume and improved neurological scores in the pMCAO model with delayed administration at 4 post-stroke hours (112), as well as decreased hemorrhage transformation related to delayed rtPA usage (117). The beneficial effects were associated with lymphopenia that sustained for at least 7 days in both peripheral circulation and the brain vasculature, as well as reduced T cell infiltration into the brain parenchyma. These beneficial effects were absent in *Rag*-/- mice (114, 115). However, one study found that fingolimod lacked protective effects on a permanent occlusion mouse model (114).

Two clinical trials have tested the effects of fingolimod on ischemic stroke. In an open-label, evaluator-blinded, parallel-group pilot trial (NCT02002390) that recruited 22 patients, oral administration of (0.5 mg) per day during the first 3 days after ischemic stroke onset in patients who could not receive alteplase treatment was well-tolerated. Moreover, it induced lymphopenia that lasted for at least 7 days, caused restricted infarct volume expansion on day 7 after stroke, and improved the 3-month neurological functions compared with the standard treatment group (118). The combined use of alteplase and fingolimod also showed protective results in both the standard (4.5 h) and delayed (4.5–6 h) time windows compared with alteplase alone, including halted lesion volume growth, decreased hemorrhage transformation, and improved 90-day neurological outcomes in a clinical trial that recruited 47 patients (119, 120). Detailed information on these clinical trials is listed in **Table 4**.

**Table 4:** Clinical Trials of T cell targeted therapies.

Study	Patients	Intervention	Control	Outcome			Safety	Study design
				Neurological function	Lesion volume	Alteplase associated adverse events		
Fingolimod								
Fu et al. (118)	22 stroke patients, anterior cerebral circulation occlusions, exceeded the 4.5h time window after stroke onset	11 patients, fingolimod plus standard management, 0.5mg/day, 3 consecutive days	11 patients, standard management	Greater reduction of NIHSS score from day 1 to day 7 Higher mBI score at 90 days Higher percentage of mRS 0-1 at 90 days	Confined enlargement of lesion volume from baseline to day 7	Not applicable	No significant difference in mortality, adverse events and infection	Open-label, evaluator-blinded, parallel-group, single center, clinical pilot trial
Zhu et al. (119)	47 stroke patients with anterior or middle cerebral arterial occlusion, received alteplase treatment within the 4.5h time window	22 patients, oral fingolimod 0.5mg/day, 3 consecutive days, plus alteplase treatment	25 patients, alteplase treatment	Greater reduction of NIHSS score on day 1 and from day 1 to day 7 Higher percentage of mRS 0-1 at 90 days	Restrained infarct expansion on day 1 and from day 1 to day 7	Smaller hemorrhage volume on day 1	No significant difference in death, adverse events, lung infection and urinary tract infection	Randomized, open-label, evaluator-blind, multicenter pilot trial
Natalizumab								
Tian et al. (120)	46 stroke patients with internal carotid artery or middle cerebral artery proximal occlusion, within 4.5 to 6 hours from stroke onset	23 patients, oral fingolimod 0.5mg/day, 3 consecutive days, plus alteplase treatment	23 patients, alteplase treatment	Higher decreased NIHSS score on day 1 Higher percentage of mRS 0-1 at 90 days	Restricted infarct expansion in baseline to 24h and 24h to 7 days	Higher percentage of asymptomatic intracranial hemorrhage, no significant difference in systematic intracranial hemorrhage	No significant difference in death and lung infection	Prospective, multicenter, randomized, open label, blinded endpoint clinical trial
Elkins et al. (121)	159 patients with acute ischemic stroke from age 18 to 85, last known normal at 6 hours or less, or 6-9h before treatment initiation, with or without alteplase	77 patients, single dose of 300mg intravenous natalizumab treatment	82 patients, 300mg intravenous placebo treatment	Significant improvement evaluated by mRS score on day 30 and BI score on day 90, changes are not significant evaluated by NIHSS score on day 30 or day 90.	No significant difference in infarct volume change on baseline to 24h, 24h to day 5, day 5 to day 30	Not applicable	Similar rates of adverse events, severe adverse events and death	Multicenter, double-blind, placebo-controlled, randomized
Elkind et al. (122)	267 supratentorial acute ischemic stroke patients, last known normal ≤9 h or >9 to ≤24 hours prior to study treatment initiation	88 patients, single dose of 300mg natalizumab, 89 patients, single dose of 600mg natalizumab	90 patients, placebo	No significant different in mRS and BI composite excellent outcome in 300 + 600mg natalizumab vs. placebo group, or in subgroups vs placebo. No significant difference in global composite excellent outcome by treatment window, tPA use or thrombectomy.	Not applicable	Not applicable	Similar rates of adverse effects and death in 300mg natalizumab group, 600mg natalizumab and placebo group.	Multicenter, double-blind, dose-ranging, placebo-controlled, randomized phase 2 study

mRS, modified Rankin Score; NIHSS, National Institutes of Health Stroke Score; mBI, modified Barthel Index.

#### Natalizumab

Natalizumab is a humanized monoclonal antibody that binds to the α4 subunit (CD49d) of the adhesion molecule α4β1 integrin (VLA-4) on lymphocytes and neutrophils. Natalizumab blocks the integrin interaction between these cells and endothelial cells, and thus prevents lymphocyte and neutrophil infiltration into the brain parenchyma (**Figure 2**). Natalizumab was approved by the FDA for treating relapse-remitting MS in 2004.

In rodent stroke models, reduced infiltration of lymphocytes and neutrophils is commonly observed in the natalizumab-treated group (30–32, 123). However, reduced infarct volume and improved behavioral outcomes are largely seen in pMCAO, but not transient stroke models (30). A preclinical randomized controlled multicenter trial confirmed that natalizumab only significantly reduced infarct volume after pMCAO while similar effects could not be seen in temporary MCAO models (123). This model-dependent effect was attributed to the magnified inflammatory response in non-reperfusion infarcts (123, 124). Contrastingly, Langhauser et al. reported that intraperitoneal administration of 300 μg natalizumab did not have any protective effect in both transient and pMCAO mouse models at day 1 and day 7 (31).

Despite the discrepancies in preclinical results, a phase II clinical trial, ACTION (NCT01955707), was initiated in 2013. This trial included 161 patients who received 300 mg of intravenous natalizumab or placebo within 9 h after stroke onset. Natalizumab treatment was negative for the primary endpoint of changes in infarct volume from day 1 to day 5; however, it improved functional outcomes as assessed by the modified Rankin Scale score (mRS) (121). To further investigate this issue, a secondary phase II clinical trial, ACTION2 (NCT02730455) was conducted. This trial enrolled 277 patients and tested the efficacy of two natalizumab doses (300 mg and 600 mg) in two different time windows (≤ 9 h or ≥ 9 h to ≤ 24 h from stroke onset). Natalizumab lacked protective effects, as measured by the mRS or Barthel Index scores, at 90 days in both doses and time windows (122). Detailed information on these clinical trials is listed in **Table 4**. This lack of long-term beneficial effects of natalizumab in clinical patients was resulted from the its transient-only lymphocyte-blocking effects and intracerebral proliferation of T cells in the delayed phase in the brain (62).

Although both fingolimod and natalizumab target T cells, their distinct results in clinical trials could further elucidate stroke pathology. First, fingolimod induces lymphopenia, which reduces lymphocyte flow to the brain vessels, while natalizumab targets adhesion molecules, which reduces T cell infiltration. This could be indicative that early detrimental effects of T cells are not dependent on their infiltration into the brain parenchyma. This “collateral damage” effect could be attributed to the secretion of soluble cytokines, as well as magnified inflammation by recruiting other immune compartments. In addition, natalizumab blocks leukocyte interaction with α4β1 integrin, whose ligand, VCAM is abundantly expressed on endothelial cells of the BBB but only on the apical side of the epithelial cells of the choroid plexus (46). The route of T cell infiltration is disease-dependent and 2/3 of all infiltrated T cells in stroke originate from the stroma of the choroid plexus, bypassing the brain-CSF barrier, while this route is not preferentially used in EAE models (35, 125). This could partially explain the failure of natalizumab in clinical stroke since it might have missed the major infiltration route. Thirdly, in clinical trials, both fingolimod and natalizumab treatments are given in the first 3 days of ischemic stroke. Although there is a lack of data on ischemic stroke, data from patients with multiple sclerosis indicate that oral administration of fingolimod typically induces a sustained lymphopenia that persists even after the discontinuation of treatment (126, 127). Natalizumab, however, only has transient effects (5 days) on blocking T cell infiltration after a single dose (62). Thus, the design used in recent clinical trials of natalizumab may have resulted in insufficient blockage of the T cell response in the brain in the chronic phase of disease. In conclusion, although post-stroke neuroinflammation and MS share mechanisms in T cell-mediated damage, their pathophysiology is fundamentally different. Future re-direction of MS treatment to ischemic stroke should take such issues into consideration.

### Treg-Targeted Therapies

Tregs exert protective roles against post-stroke inflammation. A reduction in the ratio of Tregs in the whole T cell population and Treg-secreted cytokines IL-10 and TGF-β has been reported in mice and patients (128–130). Therefore, Treg supplementation could be a potential stroke treatment (**Figure 2**).

In mouse models of stroke, the adoptive transfer of Tregs early after stroke induction led to reduced infarct volume and improved neurological function (101). Transferred T cells quickly distribute to the spleen, lymph nodes, bone marrow, liver, and blood after 24 h; however, they are not observed in the brain until 5 days after transplantation (101). This indicates that the early protective effect of transferred Tregs is not dependent on their infiltration into the parenchyma; rather, it involves inhibiting MMP production from circulating neutrophils, and therefore maintains BBB integrity (131). Consequently, there is significantly reduced infiltration of neutrophils, macrophages, and effector T cells, while no increase in the brain IL-10 levels is observed (131). The combined use of Treg transplantation and t-PA significantly reduced hemorrhage transformation and BBB dysfunction induced by delayed t-PA administration. This effect was attributed to the downregulation of CCL2 in endothelial cells (130). Tregs also act on the periphery immune system to restore immune homeostasis. After adoptive transfer of Tregs, there are reduced levels of the pro-inflammatory cytokines IL-6 and TNF-α in the blood. Notably, transferred Tregs did not mediate post-stroke immunosuppression; rather, they decreased blood bacterial loads (131).

Given the difficulty of translating the adoptive transfer of Tregs to human patients, other treatments that could stimulate Treg proliferation have been developed. Intraperitoneal injection of a CD28 superagonistic antibody in mice increases the Treg number in the peripheral blood and spleen; however, its effects on infarct size and neurological outcomes remain controversial (132, 133). Schuhmann et al. reported that the administration of this superagonist 3 days before transient stroke induction in mice significantly worsened stroke outcomes at day 1 after stroke, and this effect is independent of the presence of Tregs in the parenchyma (133). Instead, Tregs are mainly located in the vasculature. Therefore, these Tregs increase thrombus formation in the cerebral microvasculature (133). Another study employed both transient and permanent occlusion models of ischemic stroke and injected the superagonist 3 hours after stroke induction (132). This treatment increased Treg infiltration in the brain on 7 days after stroke and reduced macrophage activation. Furthermore, infarct volume and neurological function measured on day 7 revealed a protective effect (132). These discrepancies in the effects of CD28 superagonist treatment could be attributed to that prophylactic superagonist administration induces significantly higher numbers of Tregs in the circulation at stroke onset and more dominantly promoted thrombo-inflammation.

In addition, pre- or post-treatment of an IL-2/IL-2-antibody complex succeeded in reducing the infarct volume and improving neurological functions on 3 days after ischemic stroke in a Treg-dependent manner (134). Repetitive administration of this complex in the first 30 days after ischemic stroke also increased Treg counts in the periphery and the brain, and improved long-term sensorimotor functions (61). This complex boosts Treg proliferation and function through CD39/CD73 signaling, while other leukocyte groups remain largely undisturbed (134).

Bone marrow-derived stem cell (BMSC) transplantation is effective in protecting post-stroke neurological function through immunomodulation (135). Tregs are present in BMSCs; moreover, only a native proportion of Tregs within the BMSC achieved maximum neuroprotection compared with a larger or smaller proportion (136). *In vitro* studies revealed that Tregs within BMSCs increase myelin production by oligodendrocyte precursor cells, which allowed them to treat post-stroke white matter injury (137). Future studies should determine whether Tregs are the main effector cells within BMSCs and whether they interact with other cell populations in BMSCs.

### Targeting Antigen-Specific T Cell Response

Since adaptive immunity could play deleterious roles in post-stroke inflammation, treatment methods targeting autoreactive T cells have been developed. Recombinant T cell ligands (RTLs) are partial MHC class II molecule constructs comprised of the minimal TCR interface, that is the α1 and β1 domains covalently linked to specific antigen peptides (138, 139). These RTLs act as partial TCR agonists in an antigen- and species-specific manner, which inhibits T cell activation and boosts IL-10 secretion (**Figure 2**) (138, 140, 141). RTL551, which contains the α1 and β1 domains of the I-A(b) class II molecule covalently linked to the encephalitogenic MOG-35-55 peptide, reversed symptoms in experimental autoimmune encephalomyelitis mice and reduced IL-17 and TNF-α secretion from MOG-33-35 reactive CD4+ T cells (142, 143). Given that antigen-specific immunity occurs in ischemic stroke, RTL551 was initially tested in a mouse model and was found to be protective when administered at stroke onset (144) and 4 h after stroke (145, 146). This protective effect, represented by reduced infarct size at 96 h and reduced inflammatory cell infiltration, is antigen- and MHC-dependent given that it is abolished in mice treated with RTL linked to non-neural antigens or mismatched MHCs (144). The efficacy of delayed administration revealed its potential for clinical use in patients with ischemic stroke. To test its potential efficacy in human patients, a humanized recombinant T cell receptor (RTL1000), which bears human MOG-35-55, was administered to transgenic mice bearing human HLA-DR2 in a clinically relevant delayed administration time of 4 hours after stroke. RTL1000 administration decreased the infarct volume at 96 hours and reduced sensorimotor impairment 3 days after MCAO in both young and aged, male and female mouse models. Further, it exerted additive protective effects with the combined use of rt-PA (113–116). Notably, the protective effect of RTLs can be observed as early as 96 h after MCAO, when adaptive immunity against self-antigens is yet to be fully developed. This could be partially attributed to the fact that although RTLs are antigen-specific, their induction of IL-10 secretion exerts bystander immunomodulation effects. A clinical trial on the safety of RTL in MS (NCT00411723) was initiated in 2006; however, the results remain unknown. Other RTLs targeting other neural antigens and further clinical studies on the safety and efficacy of RTLs in ischemic stroke are needed.

## Conclusions

T cells are crucially involved in post-stroke inflammation. T cells adopt three different routes to infiltrate the brain parenchyma; moreover, various combinations of chemokines and their receptors on T cell subsets give spatial and temporal “address” for T cell infiltration. Early deleterious T cell responses are antigen-independent and are largely attributed to the secretion of inflammatory cytokines and their interaction with other cells to amplify the inflammation cascade. Subsequent T cell responses are antigen-dependent and manipulation of such a response could lead to immune tolerance. Tregs, which is an immunomodulatory T cell subset, allows restricted inflammation and facilitates recovery.

Treatments targeting T-cell infiltration have been developed and tested in clinical trials with mixed results. For the objective of blocking T cell infiltration, most treatments are repurposed from currently approved drugs for MS. However, the complex results from clinical trials of these treatments reveal the fundamental differences in T cell responses between these two diseases, especially in the antigen-independent phase. Future studies should consider long-term T cell responses when deciding on the dosage, timing and duration of drug administration. However, this remains a promising therapeutic direction as decreasing the number of circulating lymphocytes could be a common target, as demonstrated by the effects of fingolimod treatment. The effects of other T-cell targeted treatments for MS, such as dimethyl fumarate and teriflunomide remain to be explored. Boosting the Treg response has been shown to be effective in animal models. Antibody-mediated Treg proliferation could be a translational target, but this is yet to be tested in clinical trials. Indeed, these therapies may be limited due to the potency of Tregs to induce thrombo-inflammation in the hyperacute phase, as well as immune suppression in the peripheral immune system. Reducing the antigen specific response in ischemic stroke is also a promising target, but achieving the correct level of suppression and identifying the optimal antigen to target remain to be explored in pre-clinical models before progressing to clinical trials. In conclusion, T cells have crucial functions during post-stroke inflammation and are promising immunotherapy targets for the treatment of ischemic stroke.

## Author Contributions

DZ designed and wrote the manuscript. YL provided constructive advice on the structure of this manuscript. JR, RZ, QH, JC, Z-NG, and YY gave constructive advice and participated in proof-reading of this paper. All authors contributed to the article and approved the submitted version.

## Funding

This work was supported by the National Natural Science Foundation of China to YY (Grant No. 82071291), the Program for the Jilin Provincial Key Laboratory (20190901005JC) and the Grant from Science and Technology Department of Jilin Province (20180623052TC) to YY.

## Conflict of Interest

The authors declare that the research was conducted in the absence of any commercial or financial relationships that could be construed as a potential conflict of interest.

## References

[1] Wang YJ Li ZX Gu HQ Zhai Y Jiang Y Zhao XQ. China Stroke Statistics 2019: A Report From the National Center for Healthcare Quality Management in Neurological Diseases, China National Clinical Research Center for Neurological Diseases, the Chinese Stroke Association, National Center for Chronic and Non-Communicable Disease Control and Prevention, Chinese Center for Disease Control and Prevention and Institute for Global Neuroscience and Stroke Collaborations. Stroke Vasc Neurol (2020) 5(3):211–39. 10.1136/svn-2020-000457 PMC7548521 32826385

[2] Xu J Murphy SL Kockanek KD Arias E. Mortality in the United States, 2018. NCHS Data Brief (2020) 355):1–8. 32487294

[3] James SL Abate D Abate KH Abay SM Abbafati C Abbasi N. Global, Regional, and National Incidence, Prevalence, and Years Lived With Disability for 354 Diseases and Injuries for 195 Countries and Territories, 1990–2017: A Systematic Analysis for the Global Burden of Disease Study 2017. Lancet (2018) 392(10159):1789–858. 10.1016/S0140-6736(18)32279-7 PMC6227754 30496104

[4] Roth GA Abate D Abate KH Abay SM Abbafati C Abbasi N. Global, Regional, and National Age-Sex-Specific Mortality for 282 Causes of Death in 195 Countries and Territories, 1980–2017: A Systematic Analysis for the Global Burden of Disease Study 2017. Lancet (2018) 392(10159):1736–88. 10.1016/S0140-6736(18)32279-7 PMC6227606 30496103

[5] Krishnamurthi RV Ikeda T Feigin VL. Global, Regional and Country-Specific Burden of Ischaemic Stroke, Intracerebral Haemorrhage and Subarachnoid Haemorrhage: A Systematic Analysis of the Global Burden of Disease Study 2017. Neuroepidemiology (2020) 54(2):171–9. 10.1159/000506396 32079017

[6] Roth GA Mensah GA Johnson CO Addolorato G Ammirati E Baddour Larry M. Global Burden of Cardiovascular Diseases and Risk Factors, 1990–2019. J Am Coll Cardiol (2020) 76(25):2982–3021. 10.1016/j.jacc.2020.11.010 33309175 PMC7755038

[7] Chamorro Á Dirnagl U Urra X Planas AM. Neuroprotection in Acute Stroke: Targeting Excitotoxicity, Oxidative and Nitrosative Stress, and Inflammation. Lancet Neurol (2016) 15(8):869–81. 10.1016/s1474-4422(16)00114-9 27180033

[8] Sacks D Baxter B Campbell BCV Carpenter JS Cognard C Dippel D. Multisociety Consensus Quality Improvement Revised Consensus Statement for Endovascular Therapy of Acute Ischemic Stroke: From the American Association of Neurological Surgeons (AANS), American Society of Neuroradiology (ASNR), Cardiovascular and Interventional Radiology Society of Europe (CIRSE), Canadian Interventional Radiology Association (CIRA), Congress of Neurological Surgeons (CNS), European Society of Minimally Invasive Neurological Therapy (ESMINT), European Society of Neuroradiology (ESNR), European Stroke Organization (ESO), Society for Cardiovascular Angiography and Interventions (SCAI), Society of Interventional Radiology (SIR), Society of NeuroInterventional Surgery (SNIS), and World Stroke Organization (WSO). J Vasc Interv Radiol (2018) 29(4):441–53. 10.1016/j.jvir.2017.11.026 29478797

[9] Powers WJ Rabinstein AA Ackerson T Adeoye OM Bambakidis NC Becker K. Guidelines for the Early Management of Patients With Acute Ischemic Stroke: 2019 Update to the 2018 Guidelines for the Early Management of Acute Ischemic Stroke: A Guideline for Healthcare Professionals From the American Heart Association/American Stroke Association. Stroke (2019) 50(12):e344–418. 10.1161/STR.0000000000000211 31662037

[10] Phipps MS Cronin CA. Management of Acute Ischemic Stroke. BMJ (2020) 368:l6983. 10.1136/bmj.l6983 32054610

[11] Iadecola C Anrather J. The Immunology of Stroke: From Mechanisms to Translation. Nat Med (2011) 17(7):796–808. 10.1038/nm.2399 21738161 PMC3137275

[12] Wang Q Tang XN Yenari MA. The Inflammatory Response in Stroke. J Neuroimmunol (2007) 184(1–2):53–68. 10.1016/j.jneuroim.2006.11.014 17188755 PMC1868538

[13] Eagar TN Miller SD. 16 - Helper T-Cell Subsets and Control of the Inflammatory Response. In: Rich RR Fleisher TA Shearer WT Schroeder HW Frew AJ Weyand CM, editors. Clinical Immunology, Fifth Edition. London: Elsevier (2019). p. 235–45.e1.

[14] Nutt SL Huntington ND. 17 - Cytotoxic T Lymphocytes and Natural Killer Cells. In: Rich RR Fleisher TA Shearer WT Schroeder HW Frew AJ Weyand CM, editors. Clinical Immunology, Fifth Edition. London: Elsevier (2019). p. 247–59.e1.

[15] Gill D Veltkamp R. Dynamics of T Cell Responses After Stroke. Current Opinion in Pharmacology (2016) 26:26–32. 10.1016/j.coph.2015.09.009 26452204

[16] Kleinschnitz C Schwab N Kraft P Hagedorn I Dreykluft A Schwarz T. Early Detrimental T-Cell Effects in Experimental Cerebral Ischemia Are Neither Related to Adaptive Immunity Nor Thrombus Formation. Blood (2010) 115(18):3835–42. 10.1182/blood-2009-10-249078 20215643

[17] Yilmaz G Arumugam TV Stokes KY Granger DN. Role of T Lymphocytes and Interferon-Gamma in Ischemic Stroke. Circulation (2006) 113(17):2105–12. 10.1161/circulationaha.105.593046 16636173

[18] Liesz A Suri-Payer E Veltkamp C Doerr H Sommer C Rivest S. Regulatory T Cells Are Key Cerebroprotective Immunomodulators in Acute Experimental Stroke. Nat Med (2009) 15(2):192–9. 10.1038/nm.1927 19169263

[19] Angiari S Constantin G. Regulation of T Cell Trafficking by the T Cell Immunoglobulin and Mucin Domain 1 Glycoprotein. Trends Mol Med (2014) 20(12):675–84. 10.1016/j.molmed.2014.10.003 25457618

[20] Engelhardt B Ransohoff RM. Capture, Crawl, Cross: The T Cell Code to Breach the Blood-Brain Barriers. Trends Immunol (2012) 33(12):579–89. 10.1016/j.it.2012.07.004 22926201

[21] Zarbock A Ley K McEver RP Hidalgo A. Leukocyte Ligands for Endothelial Selectins: Specialized Glycoconjugates That Mediate Rolling and Signaling Under Flow. Blood (2011) 118(26):6743–51. 10.1182/blood-2011-07-343566 PMC3245201 22021370

[22] Ishikawa M Cooper D Russell J Salter JW Zhang JH Nanda A. Molecular Determinants of the Prothrombogenic and Inflammatory Phenotype Assumed by the Postischemic Cerebral Microcirculation. Stroke (2003) 34(7):1777–82. 10.1161/01.Str.0000074921.17767.F2 12775881

[23] Ley K Kansas GS. Selectins in T-Cell Recruitment to Non-Lymphoid Tissues and Sites of Inflammation. Nat Rev Immunol (2004) 4(5):325–36. 10.1038/nri1351 15122198

[24] Angiari S Donnarumma T Rossi B Dusi S Pietronigro E Zenaro E. TIM-1 Glycoprotein Binds the Adhesion Receptor P-Selectin and Mediates T Cell Trafficking During Inflammation and Autoimmunity. Immunity (2014) 40(4):542–53. 10.1016/j.immuni.2014.03.004 PMC4066214 24703780

[25] Matsumoto M Shigeta A Furukawa Y Tanaka T Miyasaka M Hirata T. CD43 Collaborates With P-Selectin Glycoprotein Ligand-1 to Mediate E-Selectin-Dependent T Cell Migration Into Inflamed Skin. J Immunol (2007) 178(4):2499–506. 10.4049/jimmunol.178.4.2499 17277158

[26] Alon R Feigelson SW. Chemokine-Triggered Leukocyte Arrest: Force-Regulated Bi-Directional Integrin Activation in Quantal Adhesive Contacts. Curr Opin Cell Biol (2012) 24(5):670–6. 10.1016/j.ceb.2012.06.001 22770729

[27] Engelhardt B. Immune Cell Entry Into the Central Nervous System: Involvement of Adhesion Molecules and Chemokines. J Neurol Sci (2008) 274(1-2):23–6. 10.1016/j.jns.2008.05.019 18573502

[28] Luster AD Alon R von Andrian UH. Immune Cell Migration in Inflammation: Present and Future Therapeutic Targets. Nat Immunol (2005) 6(12):1182–90. 10.1038/ni1275 16369557

[29] Schlesinger M Bendas G. Contribution of Very Late Antigen-4 (VLA-4) Integrin to Cancer Progression and Metastasis. Cancer Metastasis Rev (2015) 34(4):575–91. 10.1007/s10555-014-9545-x 25564456

[30] Liesz A Zhou W Mracsko E Karcher S Bauer H Schwarting S. Inhibition of Lymphocyte Trafficking Shields the Brain Against Deleterious Neuroinflammation After Stroke. Brain (2011) 134(Pt 3):704–20. 10.1093/brain/awr008 21354973

[31] Langhauser F Kraft P Gob E Leinweber J Schuhmann MK Lorenz K. Blocking of Alpha4 Integrin Does Not Protect From Acute Ischemic Stroke in Mice. Stroke (2014) 45(6):1799–806. 10.1161/STROKEAHA.114.005000 24743435

[32] Neumann J Riek-Burchardt M Herz J Doeppner TR Konig R Hutten H. Very-Late-Antigen-4 (VLA-4)-Mediated Brain Invasion by Neutrophils Leads to Interactions With Microglia, Increased Ischemic Injury and Impaired Behavior in Experimental Stroke. Acta Neuropathol (2015) 129(2):259–77. 10.1007/s00401-014-1355-2 25391494

[33] Kleinschnitz C Kraft P Dreykluft A Hagedorn I Göbel K Schuhmann MK. Regulatory T Cells Are Strong Promoters of Acute Ischemic Stroke in Mice by Inducing Dysfunction of the Cerebral Microvasculature. Blood (2013) 121(4):679–91. 10.1182/blood-2012-04-426734 PMC3790947 23160472

[34] Hughes CE Nibbs RJB. A Guide to Chemokines and Their Receptors. FEBS J (2018) 285(16):2944–71. 10.1111/febs.14466 PMC6120486 29637711

[35] Llovera G Benakis C Enzmann G Cai R Arzberger T Ghasemigharagoz A. The Choroid Plexus Is a Key Cerebral Invasion Route for T Cells After Stroke. Acta Neuropathol (2017) 134(6):851–68. 10.1007/s00401-017-1758-y 28762187

[36] Li P Wang L Zhou Y Gan Y Zhu W Xia Y. C-C Chemokine Receptor Type 5 (CCR5)-Mediated Docking of Transferred Tregs Protects Against Early Blood-Brain Barrier Disruption After Stroke. J Am Heart Assoc (2017) 6(8):e006387. 10.1161/jaha.117.006387 28768648 PMC5586468

[37] Ito M Komai K Mise-Omata S Iizuka-Koga M Noguchi Y Kondo T. Brain Regulatory T Cells Suppress Astrogliosis and Potentiate Neurological Recovery. Nature (2019) 565(7738):246–50. 10.1038/s41586-018-0824-5 30602786

[38] Arunachalam P Ludewig P Melich P Arumugam TV Gerloff C Prinz I. CCR6 (CC Chemokine Receptor 6) Is Essential for the Migration of Detrimental Natural Interleukin-17-Producing Gammadelta T Cells in Stroke. Stroke (2017) 48(7):1957–65. 10.1161/STROKEAHA.117.016753 28611085

[39] Benakis C Llovera G Liesz A. The Meningeal and Choroidal Infiltration Routes for Leukocytes in Stroke. Ther Adv Neurol Disord (2018) 11:1756286418783708. 10.1177/1756286418783708 29977343 PMC6024265

[40] Hoffmann A Dege T Kunze R Ernst AS Lorenz H Böhler LI. Early Blood-Brain Barrier Disruption in Ischemic Stroke Initiates Multifocally Around Capillaries/Venules. Stroke (2018) 49(6):1479–87. 10.1161/strokeaha.118.020927 29760276

[41] Giraud M Cho TH Nighoghossian N Maucort-Boulch D Deiana G Østergaard L. Early Blood Brain Barrier Changes in Acute Ischemic Stroke: A Sequential MRI Study. J Neuroimaging (2015) 25(6):959–63. 10.1111/jon.12225 25702824

[42] Jiang X Andjelkovic AV Zhu L Yang T Bennett MVL Chen J. Blood-Brain Barrier Dysfunction and Recovery After Ischemic Stroke. Prog Neurobiol (2018) 163-164:144–71. 10.1016/j.pneurobio.2017.10.001 PMC5886838 28987927

[43] Yang C Hawkins KE Doré S Candelario-Jalil E. Neuroinflammatory Mechanisms of Blood-Brain Barrier Damage in Ischemic Stroke. Am J Physiol Cell Physiol (2019) 316(2):C135–c53. 10.1152/ajpcell.00136.2018 PMC6397344 30379577

[44] Yang Y Rosenberg GA. Blood-Brain Barrier Breakdown in Acute and Chronic Cerebrovascular Disease. Stroke (2011) 42(11):3323–8. 10.1161/strokeaha.110.608257 PMC3584169 21940972

[45] Lauer AN Tenenbaum T Schroten H Schwerk C. The Diverse Cellular Responses of the Choroid Plexus During Infection of the Central Nervous System. Am J Physiol Cell Physiol (2018) 314(2):C152–C65. 10.1152/ajpcell.00137.2017 29070490

[46] Steffen BJ Breier G Butcher EC Schulz M Engelhardt B. ICAM-1, VCAM-1, and MAdCAM-1 Are Expressed on Choroid Plexus Epithelium But Not Endothelium and Mediate Binding of Lymphocytes *in vitro* . Am J Pathol (1996) 148(6):1819–38. PMC1861637 8669469

[47] Miró-Mur F Urra X Ruiz-Jaén F Pedragosa J Chamorro Á Planas AM. Antigen-Dependent T Cell Response to Neural Peptides After Human Ischemic Stroke. Front Cell Neurosci (2020) 14:206. 10.3389/fncel.2020.00206 32719588 PMC7348665

[48] Benakis C Brea D Caballero S Faraco G Moore J Murphy M. Commensal Microbiota Affects Ischemic Stroke Outcome by Regulating Intestinal Gammadelta T Cells. Nat Med (2016) 22(5):516–23. 10.1038/nm.4068 PMC4860105 27019327

[49] Alves de Lima K Rustenhoven J Da Mesquita S Wall M Salvador AF Smirnov I. Meningeal γδ T Cells Regulate Anxiety-Like Behavior *Via* IL-17a Signaling in Neurons. Nat Immunol (2020) 21(11):1421–9. 10.1038/s41590-020-0776-4 PMC8496952 32929273

[50] Gelderblom M Weymar A Bernreuther C Velden J Arunachalam P Steinbach K. Neutralization of the IL-17 Axis Diminishes Neutrophil Invasion and Protects From Ischemic Stroke. Blood (2012) 120(18):3793–802. 10.1182/blood-2012-02-412726 22976954

[51] Gronberg NV Johansen FF Kristiansen U Hasseldam H. Leukocyte Infiltration in Experimental Stroke. J Neuroinflamm (2013) 10:115. 10.1186/1742-2094-10-115 PMC3852747 24047275

[52] Gelderblom M Leypoldt F Steinbach K Behrens D Choe CU Siler DA. Temporal and Spatial Dynamics of Cerebral Immune Cell Accumulation in Stroke. Stroke (2009) 40(5):1849–57. 10.1161/strokeaha.108.534503 19265055

[53] Shichita T Sugiyama Y Ooboshi H Sugimori H Nakagawa R Takada I. Pivotal Role of Cerebral Interleukin-17-Producing gammadeltaT Cells in the Delayed Phase of Ischemic Brain Injury. Nat Med (2009) 15(8):946–50. 10.1038/nm.1999 19648929

[54] Takata M Nakagomi T Kashiwamura S Nakano-Doi A Saino O Nakagomi N. Glucocorticoid-Induced TNF Receptor-Triggered T Cells Are Key Modulators for Survival/Death of Neural Stem/Progenitor Cells Induced by Ischemic Stroke. Cell Death Differ (2012) 19(5):756–67. 10.1038/cdd.2011.145 PMC3321616 22052192

[55] Vindegaard N Munoz-Briones C El Ali HH Kristensen LK Rasmussen RS Johansen FF. T-Cells and Macrophages Peak Weeks After Experimental Stroke: Spatial and Temporal Characteristics. Neuropathology (2017) 37(5):407–14. 10.1111/neup.12387 28517732

[56] Xie L Li W Hersh J Liu R Yang SH. Experimental Ischemic Stroke Induces Long-Term T Cell Activation in the Brain. J Cereb Blood Flow Metab (2019) 39(11):2268–76. 10.1177/0271678X18792372 PMC6827125 30092705

[57] Liesz A Karcher S Veltkamp R. Spectratype Analysis of Clonal T Cell Expansion in Murine Experimental Stroke. J Neuroimmunol (2013) 257(1–2):46–52. 10.1016/j.jneuroim.2013.01.013 23498140

[58] Li GZ Zhong D Yang LM Sun B Zhong ZH Yin YH. Expression of Interleukin-17 in Ischemic Brain Tissue. Scand J Immunol (2005) 62(5):481–6. 10.1111/j.1365-3083.2005.01683.x 16305645

[59] Stubbe T Ebner F Richter D Engel OR Klehmet J Royl G. Regulatory T Cells Accumulate and Proliferate in the Ischemic Hemisphere for Up to 30 Days After MCAO. J Cereb Blood Flow Metab (2012) 33(1):37–47. 10.1038/jcbfm.2012.128 22968321 PMC3597367

[60] Meng H Zhao H Cao X Hao J Zhang H Liu Y. Double-Negative T Cells Remarkably Promote Neuroinflammation After Ischemic Stroke. Proc Natl Acad Sci (2019) 116(12):5558–63. 10.1073/pnas.1814394116 PMC6431175 30819895

[61] Shi L Sun Z Su W Xu F Xie D Zhang Q. Treg Cell-Derived Osteopontin Promotes Microglia-Mediated White Matter Repair After Ischemic Stroke. Immunity (2021). 10.1016/j.immuni.2021.04.022 PMC8282725 34015256

[62] Heindl S Ricci A Carofiglio O Zhou Q Arzberger T Lenart N. Chronic T Cell Proliferation in Brains After Stroke Could Interfere With the Efficacy of Immunotherapies. J Experiment Med (2021) 218(8):e20202411. 10.1084/jem.20202411 PMC8160576 34037669

[63] Kollikowski AM Schuhmann MK Nieswandt B Mullges W Stoll G Pham M. Local Leukocyte Invasion During Hyperacute Human Ischemic Stroke. Ann Neurol (2020) 87(3):466–79. 10.1002/ana.25665 31899551

[64] Jenkins MK Khoruts A Ingulli E Mueller DL McSorley SJ Reinhardt RL. *In Vivo* Activation of Antigen-Specific CD4 T Cells. Annu Rev Immunol (2001) 19(1):23–45. 10.1146/annurev.immunol.19.1.23 11244029

[65] Weaver CT Hatton RD Mangan PR Harrington LE. IL-17 Family Cytokines and the Expanding Diversity of Effector T Cell Lineages. Annu Rev Immunol (2007) 25:821–52. 10.1146/annurev.immunol.25.022106.141557 17201677

[66] Chaitanya GV Eeka P Munker R Alexander JS Babu PP. Role of Cytotoxic Protease Granzyme-B in Neuronal Degeneration During Human Stroke. Brain Pathol (2011) 21(1):16–30. 10.1111/j.1750-3639.2010.00426.x 20825413 PMC8094313

[67] Loetscher P Pellegrino A Gong JH Mattioli I Loetscher M Bardi G. The Ligands of CXC Chemokine Receptor 3, I-TAC, Mig, and IP10, Are Natural Antagonists for CCR3. J Biol Chem (2001) 276(5):2986–91. 10.1074/jbc.M005652200 11110785

[68] Clarkson BD Ling C Shi Y Harris MG Rayasam A Sun D. T Cell-Derived Interleukin (IL)-21 Promotes Brain Injury Following Stroke in Mice. J Exp Med (2014) 211(4):595–604. 10.1084/jem.20131377 24616379 PMC3978271

[69] Lee HK Keum S Sheng H Warner DS Lo DC Marchuk DA. Natural Allelic Variation of the IL-21 Receptor Modulates Ischemic Stroke Infarct Volume. J Clin Invest (2016) 126(8):2827–38. 10.1172/JCI84491 PMC4966306 27400126

[70] Schuhmann MK Stoll G Bieber M Vogtle T Hofmann S Klaus V. CD84 Links T Cell and Platelet Activity in Cerebral Thrombo-Inflammation in Acute Stroke. Circulation Res (2020) 127(8):1023–35. 10.1161/CIRCRESAHA.120.316655 PMC7508294 32762491

[71] Wang J Xie L Yang C Ren C Zhou K Wang B. Activated Regulatory T Cell Regulates Neural Stem Cell Proliferation in the Subventricular Zone of Normal and Ischemic Mouse Brain Through Interleukin 10. Front Cell Neurosci (2015) 9:361. 10.3389/fncel.2015.00361 26441532 PMC4568339

[72] Li P Mao L Liu X Gan Y Zheng J Thomson AW. Essential Role of Program Death 1-Ligand 1 in Regulatory T-Cell-Afforded Protection Against Blood-Brain Barrier Damage After Stroke. Stroke (2014) 45(3):857–64. 10.1161/strokeaha.113.004100 PMC3939692 24496394

[73] Mracsko E Liesz A Stojanovic A Lou WPK Osswald M Zhou W. Antigen Dependently Activated Cluster of Differentiation 8-Positive T Cells Cause Perforin-Mediated Neurotoxicity in Experimental Stroke. J Neurosci (2014) 34(50):16784–95. 10.1523/jneurosci.1867-14.2014 PMC6608504 25505331

[74] Cserr HF Harling-Berg CJ Knopf PM. Drainage of Brain Extracellular Fluid Into Blood and Deep Cervical Lymph and its Immunological Significance. Brain Pathol (1992) 2(4):269–76. 10.1111/j.1750-3639.1992.tb00703.x 1341962

[75] Carare RO Hawkes CA Weller RO. Afferent and Efferent Immunological Pathways of the Brain. Anatomy, Function and Failure. Brain Behav Immun (2014) 36:9–14. 10.1016/j.bbi.2013.10.012 24145049

[76] Miro-Mur F Urra X Gallizioli M Chamorro A Planas AM. Antigen Presentation After Stroke. Neurotherapeutics (2016) 13(4):719–28. 10.1007/s13311-016-0469-8 PMC5081119 27514673

[77] Planas AM Gómez-Choco M Urra X Gorina R Caballero M Chamorro Á. Brain-Derived Antigens in Lymphoid Tissue of Patients With Acute Stroke. J Immunol (2012) 188(5):2156–63. 10.4049/jimmunol.1102289 22287710

[78] Jin WN Gonzales R Feng Y Wood K Chai Z Dong JF. Brain Ischemia Induces Diversified Neuroantigen-Specific T-Cell Responses That Exacerbate Brain Injury. Stroke (2018) 49(6):1471–8. 10.1161/STROKEAHA.118.020203 PMC5976228 29695462

[79] Rustenhoven J Drieu A Mamuladze T de Lima KA Dykstra T Wall M. Functional Characterization of the Dural Sinuses as a Neuroimmune Interface. Cell (2021) 184(4):1000–16.e27. 10.1016/j.cell.2020.12.040 33508229 PMC8487654

[80] Lutz MB Kurts C. Induction of Peripheral CD4+ T-Cell Tolerance and CD8+ T-Cell Cross-Tolerance by Dendritic Cells. Eur J Immunol (2009) 39(9):2325–30. 10.1002/eji.200939548 19701895

[81] Becker KJ McCarron RM Ruetzler C Laban O Sternberg E Flanders KC. Immunologic Tolerance to Myelin Basic Protein Decreases Stroke Size After Transient Focal Cerebral Ischemia. Proc Natl Acad Sci U S A (1997) 94(20):10873–8. 10.1073/pnas.94.20.10873 PMC23514 9380727

[82] Becker KJ. Sensitization and Tolerization to Brain Antigens in Stroke. Neuroscience (2009) 158(3):1090–7. 10.1016/j.neuroscience.2008.07.027 PMC2684331 18706487

[83] Chen Y Ruetzler C Pandipati S Spatz M McCarron RM Becker K. Mucosal Tolerance to E-Selectin Provides Cell-Mediated Protection Against Ischemic Brain Injury. Proc Natl Acad Sci U S A (2003) 100(25):15107–12. 10.1073/pnas.2436538100 PMC299916 14645708

[84] Schuhmann MK Langhauser F Kraft P Kleinschnitz C. B Cells do Not Have a Major Pathophysiologic Role in Acute Ischemic Stroke in Mice. J Neuroinflamm (2017) 14(1):112. 10.1186/s12974-017-0890-x PMC5457733 28576128

[85] Seifert HA Collier LA Chapman CB Benkovic SA Willing AE Pennypacker KR. Pro-Inflammatory Interferon Gamma Signaling Is Directly Associated With Stroke Induced Neurodegeneration. J Neuroimmune Pharmacol (2014) 9(5):679–89. 10.1007/s11481-014-9560-2 PMC4209188 25104571

[86] Gelderblom M Gallizioli M Ludewig P Thom V Arunachalam P Rissiek B. IL-23 (Interleukin-23)-Producing Conventional Dendritic Cells Control the Detrimental IL-17 (Interleukin-17) Response in Stroke. Stroke (2018) 49(1):155–64. 10.1161/STROKEAHA.117.019101 29212740

[87] Zheng Y Zhong D Chen H Ma S Sun Y Wang M. Pivotal Role of Cerebral Interleukin-23 During Immunologic Injury in Delayed Cerebral Ischemia in Mice. Neuroscience (2015) 290:321–31. 10.1016/j.neuroscience.2015.01.041 25637493

[88] Meares GP Ma X Qin H Benveniste EN. Regulation of CCL20 Expression in Astrocytes by IL-6 and IL-17. Glia (2012) 60(5):771–81. 10.1002/glia.22307 22319003

[89] Wang DD Zhao YF Wang GY Sun B Kong QF Zhao K. IL-17 Potentiates Neuronal Injury Induced by Oxygen-Glucose Deprivation and Affects Neuronal IL-17 Receptor Expression. J Neuroimmunol (2009) 212(1-2):17–25. 10.1016/j.jneuroim.2009.04.007 19457561

[90] Kawanokuchi J Shimizu K Nitta A Yamada K Mizuno T Takeuchi H. Production and Functions of IL-17 in Microglia. J Neuroimmunol (2008) 194(1):54–61. 10.1016/j.jneuroim.2007.11.006 18164424

[91] Stoll G Nieswandt B. Thrombo-Inflammation in Acute Ischaemic Stroke - Implications for Treatment. Nat Rev Neurol (2019) 15(8):473–81. 10.1038/s41582-019-0221-1 31263257

[92] Schuhmann MK Bieber M Franke M Kollikowski AM Stegner D Heinze KG. Platelets and Lymphocytes Drive Progressive Penumbral Tissue Loss During Middle Cerebral Artery Occlusion in Mice. J Neuroinflamm (2021) 18(1):46. 10.1186/s12974-021-02095-1 PMC7890632 33602266

[93] Denorme F Martinod K Vandenbulcke A Denis CV Lenting PJ Deckmyn H. The Von Willebrand Factor A1 Domain Mediates Thromboinflammation, Aggravating Ischemic Stroke Outcome in Mice. Haematologica (2020) 106(3):819–28. 10.3324/haematol.2019.241042 PMC7927893 32107335

[94] Schuhmann MK Guthmann J Stoll G Nieswandt B Kraft P Kleinschnitz C. Blocking of Platelet Glycoprotein Receptor Ib Reduces “Thrombo-Inflammation” in Mice With Acute Ischemic Stroke. J Neuroinflamm (2017) 14(1):18. 10.1186/s12974-017-0792-y PMC5251224 28109273

[95] Voskoboinik I Dunstone MA Baran K Whisstock JC Trapani JA. Perforin: Structure, Function, and Role in Human Immunopathology. Immunol Rev (2010) 235(1):35–54. 10.1111/j.0105-2896.2010.00896.x 20536554

[96] Voskoboinik I Whisstock JC Trapani JA. Perforin and Granzymes: Function, Dysfunction and Human Pathology. Nat Rev Immunol (2015) 15(6):388–400. 10.1038/nri3839 25998963

[97] Selvaraj UM Ujas TA Kong X Kumar A Plautz EJ Zhang S. Delayed Egress of CD8 T Cells Contributes to Long-Term Pathology After Ischemic Stroke in Male Mice. Brain Behav Immun (2021) 95:502–13. 10.1016/j.bbi.2021.05.001 PMC8221572 33964435

[98] Chaitanya GV Schwaninger M Alexander JS Babu PP. Granzyme-B Is Involved in Mediating Post-Ischemic Neuronal Death During Focal Cerebral Ischemia in Rat Model. Neuroscience (2010) 165(4):1203–16. 10.1016/j.neuroscience.2009.10.067 19895873

[99] Chen W Jin W Hardegen N Lei KJ Li L Marinos N. Conversion of Peripheral CD4+CD25- Naive T Cells to CD4+CD25+ Regulatory T Cells by TGF-Beta Induction of Transcription Factor Foxp3. J Exp Med (2003) 198(12):1875–86. 10.1084/jem.20030152 PMC2194145 14676299

[100] Meng X Yang J Dong M Zhang K Tu E Gao Q. Regulatory T Cells in Cardiovascular Diseases. Nat Rev Cardiol (2016) 13(3):167–79. 10.1038/nrcardio.2015.169 PMC11849084 26525543

[101] Li P Gan Y Sun B-L Zhang F Lu B Gao Y. Adoptive Regulatory T-Cell Therapy Protects Against Cerebral Ischemia. Ann Neurol (2013) 74(3):458–71. 10.1002/ana.23815 PMC3748165 23674483

[102] Liesz A Zhou W Na SY Hämmerling GJ Garbi N Karcher S. Boosting Regulatory T Cells Limits Neuroinflammation in Permanent Cortical Stroke. J Neurosci (2013) 33(44):17350–62. 10.1523/jneurosci.4901-12.2013 PMC6618366 24174668

[103] Liesz A Bauer A Hoheisel JD Veltkamp R. Intracerebral Interleukin-10 Injection Modulates Post-Ischemic Neuroinflammation: An Experimental Microarray Study. Neurosci Lett (2014) 579:18–23. 10.1016/j.neulet.2014.07.003 25019688

[104] Protti GG Gagliardi RJ Forte WC Sprovieri SR. Interleukin-10 may Protect Against Progressing Injury During the Acute Phase of Ischemic Stroke. Arq Neuropsiquiatr (2013) 71(11):846–51. 10.1590/0004-282x20130168 24394869

[105] Anderson MF Blomstrand F Blomstrand C Eriksson PS Nilsson M. Astrocytes and Stroke: Networking for Survival? Neurochem Res (2003) 28(2):293–305. 10.1023/a:1022385402197 12608702

[106] Jin K Sun Y Xie L Peel A Mao XO Batteur S. Directed Migration of Neuronal Precursors Into the Ischemic Cerebral Cortex and Striatum. Mol Cell Neurosci (2003) 24(1):171–89. 10.1016/S1044-7431(03)00159-3 14550778

[107] Yamashita T Ninomiya M Hernández Acosta P García-Verdugo JM Sunabori T Sakaguchi M. Subventricular Zone-Derived Neuroblasts Migrate and Differentiate Into Mature Neurons in the Post-Stroke Adult Striatum. J Neurosci (2006) 26(24):6627. 10.1523/JNEUROSCI.0149-06.2006 16775151 PMC6674034

[108] Chun J Kihara Y Jonnalagadda D Blaho VA. Fingolimod: Lessons Learned and New Opportunities for Treating Multiple Sclerosis and Other Disorders. Annu Rev Pharmacol Toxicol (2019) 59:149–70. 10.1146/annurev-pharmtox-010818-021358 PMC6392001 30625282

[109] Mandala S Hajdu R Bergstrom J Quackenbush E Xie J Milligan J. Alteration of Lymphocyte Trafficking by Sphingosine-1-Phosphate Receptor Agonists. Science (2002) 296(5566):346–9. 10.1126/science.1070238 11923495

[110] Wang Z Kawabori M Houkin K. FTY720 (Fingolimod) Ameliorates Brain Injury Through Multiple Mechanisms and Is a Strong Candidate for Stroke Treatment. Curr Med Chem (2020) 27(18):2979–93. 10.2174/0929867326666190308133732 PMC7403647 31785606

[111] Matloubian M Lo CG Cinamon G Lesneski MJ Xu Y Brinkmann V. Lymphocyte Egress From Thymus and Peripheral Lymphoid Organs Is Dependent on S1P Receptor 1. Nature (2004) 427(6972):355–60. 10.1038/nature02284 14737169

[112] Wei Y Yemisci M Kim HH Yung LM Shin HK Hwang SK. Fingolimod Provides Long-Term Protection in Rodent Models of Cerebral Ischemia. Ann Neurol (2011) 69(1):119–29. 10.1002/ana.22186 PMC3200194 21280082

[113] Hasegawa Y Suzuki H Sozen T Rolland W Zhang JH. Activation of Sphingosine 1-Phosphate Receptor-1 by FTY720 Is Neuroprotective After Ischemic Stroke in Rats. Stroke (2010) 41(2):368–74. 10.1161/STROKEAHA.109.568899 PMC2811754 19940275

[114] Liesz A Sun L Zhou W Schwarting S Mracsko E Zorn M. FTY720 Reduces Post-Ischemic Brain Lymphocyte Influx But Does Not Improve Outcome in Permanent Murine Cerebral Ischemia. PloS One (2011) 6(6):e21312. 10.1371/journal.pone.0021312 21701599 PMC3119049

[115] Kraft P Göb E Schuhmann MK Göbel K Deppermann C Thielmann I. FTY720 Ameliorates Acute Ischemic Stroke in Mice by Reducing Thrombo-Inflammation But Not by Direct Neuroprotection. Stroke (2013) 44(11):3202–10. 10.1161/strokeaha.113.002880 24029635

[116] Dang C Lu Y Li Q Wang C Ma X. Efficacy of the Sphingosine-1-Phosphate Receptor Agonist Fingolimod in Animal Models of Stroke: An Updated Meta-Analysis. Int J Neurosci (2020) 131(1):85–94. 10.1080/00207454.2020.1733556 32148137

[117] Campos F Qin T Castillo J Seo JH Arai K Lo EH. Fingolimod Reduces Hemorrhagic Transformation Associated With Delayed Tissue Plasminogen Activator Treatment in a Mouse Thromboembolic Model. Stroke (2013) 44(2):505–11. 10.1161/strokeaha.112.679043 PMC3586809 23287783

[118] Fu Y Zhang N Ren L Yan Y Sun N Li YJ. Impact of an Immune Modulator Fingolimod on Acute Ischemic Stroke. Proc Natl Acad Sci U S A (2014) 111(51):18315–20. 10.1073/pnas.1416166111 PMC4280578 25489101

[119] Zhu Z Fu Y Tian D Sun N Han W Chang G. Combination of the Immune Modulator Fingolimod With Alteplase in Acute Ischemic Stroke: A Pilot Trial. Circulation (2015) 132(12):1104–12. 10.1161/circulationaha.115.016371 PMC4580515 26202811

[120] Tian DC Shi K Zhu Z Yao J Yang X Su L. Fingolimod Enhances the Efficacy of Delayed Alteplase Administration in Acute Ischemic Stroke by Promoting Anterograde Reperfusion and Retrograde Collateral Flow. Ann Neurol (2018) 84(5):717–28. 10.1002/ana.25352 PMC6282815 30295338

[121] Elkins J Veltkamp R Montaner J Johnston SC Singhal AB Becker K. Safety and Efficacy of Natalizumab in Patients With Acute Ischaemic Stroke (ACTION): A Randomised, Placebo-Controlled, Double-Blind Phase 2 Trial. Lancet Neurol (2017) 16(3):217–26. 10.1016/s1474-4422(16)30357-x 28229893

[122] Elkind MSV Veltkamp R Montaner J Johnston SC Singhal AB Becker K. Natalizumab in Acute Ischemic Stroke (ACTION II): A Randomized, Placebo-Controlled Trial. Neurology (2020) 95(8):e1091–e104. 10.1212/wnl.0000000000010038 PMC7668547 32591475

[123] Llovera G Hofmann K Roth S Salas-Pérdomo A Ferrer-Ferrer M Perego C. Results of a Preclinical Randomized Controlled Multicenter Trial (pRCT): Anti-CD49d Treatment for Acute Brain Ischemia. Sci Transl Med (2015) 7(299):299ra121. 10.1126/scitranslmed.aaa9853 26246166

[124] Chu HX Kim HA Lee S Moore JP Chan CT Vinh A. Immune Cell Infiltration in Malignant Middle Cerebral Artery Infarction: Comparison With Transient Cerebral Ischemia. J Cereb Blood Flow Metab (2014) 34(3):450–9. 10.1038/jcbfm.2013.217 PMC3948121 24326388

[125] Schläger C Körner H Krueger M Vidoli S Haberl M Mielke D. Effector T-Cell Trafficking Between the Leptomeninges and the Cerebrospinal Fluid. Nature (2016) 530(7590):349–53. 10.1038/nature16939 26863192

[126] Chitnis T Banwell B Krupp L Arnold DL Bar-Or A Brück W. Temporal Profile of Lymphocyte Counts and Relationship With Infections With Fingolimod Therapy in Paediatric Patients With Multiple Sclerosis: Results From the PARADIGMS Study. Mult Scler (2021) 27(6):922–32. 10.1177/1352458520936934 32633694

[127] Nagy S Kuhle J Derfuss T. Lymphocyte Recovery After Fingolimod Discontinuation in Patients With MS. Neurol Neuroimmunol Neuroinflamm (2020) 7(6):e874. 10.1212/nxi.0000000000000874 32801166 PMC7641093

[128] Ruhnau J Schulze J von Sarnowski B Heinrich M Langner S Pötschke C. Reduced Numbers and Impaired Function of Regulatory T Cells in Peripheral Blood of Ischemic Stroke Patients. Mediators Inflamm (2016) 2016:2974605. 10.1155/2016/2974605 27073295 PMC4814689

[129] Hu Y Zheng Y Wu Y Ni B Shi S. Imbalance Between IL-17A-Producing Cells and Regulatory T Cells During Ischemic Stroke. Mediators Inflamm (2014) 2014:813045. 10.1155/2014/813045 24991091 PMC4058812

[130] Mao L Li P Zhu W Cai W Liu Z Wang Y. Regulatory T Cells Ameliorate Tissue Plasminogen Activator-Induced Brain Haemorrhage After Stroke. Brain (2017) 140(7):1914–31. 10.1093/brain/awx111 PMC6059175 28535201

[131] Li P Mao L Zhou G Leak RK Sun BL Chen J. Adoptive Regulatory T-Cell Therapy Preserves Systemic Immune Homeostasis After Cerebral Ischemia. Stroke (2013) 44(12):3509–15. 10.1161/STROKEAHA.113.002637 PMC3895539 24092548

[132] Na SY Mracsko E Liesz A Hünig T Veltkamp R. Amplification of Regulatory T Cells Using a CD28 Superagonist Reduces Brain Damage After Ischemic Stroke in Mice. Stroke (2015) 46(1):212–20. 10.1161/strokeaha.114.007756 25378432

[133] Schuhmann MK Kraft P Stoll G Lorenz K Meuth SG Wiendl H. CD28 Superagonist-Mediated Boost of Regulatory T Cells Increases Thrombo-Inflammation and Ischemic Neurodegeneration During the Acute Phase of Experimental Stroke. J Cereb Blood Flow Metab (2015) 35(1):6–10. 10.1038/jcbfm.2014.175 25315859 PMC4294400

[134] Zhang H Xia Y Ye Q Yu F Zhu W Li P. In Vivo Expansion of Regulatory T Cells With IL-2/IL-2 Antibody Complex Protects Against Transient Ischemic Stroke. J Neurosci (2018) 38(47):10168–79. 10.1523/jneurosci.3411-17.2018 PMC6246882 30291203

[135] Wang LQ Lin ZZ Zhang HX Shao B Xiao L Jiang HG. Timing and Dose Regimens of Marrow Mesenchymal Stem Cell Transplantation Affect the Outcomes and Neuroinflammatory Response After Ischemic Stroke. CNS Neurosci Ther (2014) 20(4):317–26. 10.1111/cns.12216 PMC6493061 24393245

[136] Neal EG Acosta SA Kaneko Y Ji X Borlongan CV. Regulatory T-Cells Within Bone Marrow-Derived Stem Cells Actively Confer Immunomodulatory and Neuroprotective Effects Against Stroke. J Cereb Blood Flow Metab (2019) 39(9):1750–8. 10.1177/0271678X18766172 PMC6727132 29569981

[137] Zarriello S Neal EG Kaneko Y Borlongan CV. T-Regulatory Cells Confer Increased Myelination and Stem Cell Activity After Stroke-Induced White Matter Injury. J Clin Med (2019) 8(4):537. 10.3390/jcm8040537 PMC6518209 31010132

[138] Huan J Subramanian S Jones R Rich C Link J Mooney J. Monomeric Recombinant TCR Ligand Reduces Relapse Rate and Severity of Experimental Autoimmune Encephalomyelitis in SJL/J Mice Through Cytokine Switch. J Immunol (2004) 172(7):4556–66. 10.4049/jimmunol.172.7.4556 15034073

[139] Benedek G Vandenbark AA Alkayed NJ Offner H. Partial MHC Class II Constructs as Novel Immunomodulatory Therapy for Stroke. Neurochem Int (2017) 107:138–47. 10.1016/j.neuint.2016.10.007 PMC5411346 27773790

[140] Burrows GG Chou YK Wang C Chang JW Finn TP Culbertson NE. Rudimentary TCR Signaling Triggers Default IL-10 Secretion by Human Th1 Cells. J Immunol (2001) 167(8):4386–95. 10.4049/jimmunol.167.8.4386 11591763

[141] Wang C Mooney JL Meza-Romero R Chou YK Huan J Vandenbark AA. Recombinant TCR Ligand Induces Early TCR Signaling and a Unique Pattern of Downstream Activation. J Immunol (2003) 171(4):1934–40. 10.4049/jimmunol.171.4.1934 12902496

[142] Sinha S Subramanian S Emerson-Webber A Lindner M Burrows GG Grafe M. Recombinant TCR Ligand Reverses Clinical Signs and CNS Damage of EAE Induced by Recombinant Human MOG. J Neuroimmune Pharmacol (2010) 5(2):231–9. 10.1007/s11481-009-9175-1 PMC2866769 19789980

[143] Sinha S Subramanian S Proctor TM Kaler LJ Grafe M Dahan R. A Promising Therapeutic Approach for Multiple Sclerosis: Recombinant T-Cell Receptor Ligands Modulate Experimental Autoimmune Encephalomyelitis by Reducing Interleukin-17 Production and Inhibiting Migration of Encephalitogenic Cells Into the CNS. J Neurosci (2007) 27(46):12531–9. 10.1523/jneurosci.3599-07.2007 PMC6673319 18003831

[144] Subramanian S Zhang B Kosaka Y Burrows GG Grafe MR Vandenbark AA. Recombinant T Cell Receptor Ligand Treats Experimental Stroke. Stroke (2009) 40(7):2539–45. 10.1161/strokeaha.108.543991 PMC2704258 19443805

[145] Akiyoshi K Dziennis S Palmateer J Ren X Vandenbark AA Offner H. Recombinant T Cell Receptor Ligands Improve Outcome After Experimental Cerebral Ischemia. Transl Stroke Res (2011) 2(3):404–10. 10.1007/s12975-011-0085-1 PMC3181103 21961027

[146] Dziennis S Mader S Akiyoshi K Ren X Ayala P Burrows GG. Therapy With Recombinant T-Cell Receptor Ligand Reduces Infarct Size and Infiltrating Inflammatory Cells in Brain After Middle Cerebral Artery Occlusion in Mice. Metab Brain Dis (2011) 26(2):123–33. 10.1007/s11011-011-9241-2 PMC3111858 21472429

